# Advanced Flexible Bioelectrodes for Next‐Generation Implantable Triboelectric Nanogenerators

**DOI:** 10.1155/ijbm/5695793

**Published:** 2026-04-27

**Authors:** Viraj P. Nirwan, Altangerel Amarjargal, Viktorie Ročková, Martin Timusk, Linards Lapčinskis, Rebecca Hengsbach, Eva Filová, Andris Šutka, Amir Fahmi

**Affiliations:** ^1^ Faculty of Technology and Bionics, Rhine-Waal University of Applied Sciences, Marie-Curie-Straße 1, Kleve, 47533, Germany, hochschule-rhein-waal.de; ^2^ Institute of Experimental Medicine of the Czech Academy of Sciences, Vídeňská 1083, Prague, 14220, Czech Republic, cas.cz; ^3^ Department of Physiology, Faculty of Science, Charles University in Prague, Viničná 7, Praha, 12800, Czech Republic, cuni.cz; ^4^ Institute of Physics, University of Tartu, W. Ostwaldi Str. 1, Tartu, 50411, Estonia, ut.ee; ^5^ Institute of Physics and Materials Science, Faculty of Natural Sciences and Technology, Riga Technical University, P. Valdena Street 3, Riga, LV1048, Latvia, rtu.lv

**Keywords:** biocompatible polymers, electrospun hybrid nanofibers, TENG-powered capacitors, triboelectric nanogenerators

## Abstract

The fabrication of novel bioelectrodes using electrospun nanofibers and a prototype for a sustainable triboelectric nanogenerator (TENG) is explored in this study. Poly(lactic acid‐caprolactone) (PLCL) was electrospun as the matrix of the bioelectrode and functionalized with a commercial graphene XT3. Afterward, the fibers were coated with graphene ink (Gr.ink) or poly(3,4‐ethylenedioxythiophene)‐poly(styrene sulfonate) (PEDOT:PSS). The average diameter of nanofibers increased multifold after coating. PEDOT:PSS‐coated fibers showed the highest Young’s modulus at 98 MPa. The nanofiber mats did not show decreased metabolic activity below the cytotoxic threshold. Graphene‐functionalized PLCL fiber coated with Gr.ink showed a significant decrease in proliferation compared to untreated cells. The tested mats did not support human dermal cells’ adhesion. The nanofibers blended with graphene and coated with PEDOT:PSS showed the highest conductivity, sufficient for use in TENG devices. A TENG device was assembled using PEDOT:PSS‐covered PLCL/graphene fiber mats as electrodes and poly(lactic acid) with poly(glycerol sebacate) as active contact layers. The TENG device achieved a power density of 1.9 mW m^−2^ and, during 1 min of operation, charged the capacitor to a voltage corresponding to 71 nJ of stored energy. The TENG module proposed could address the energy demands of healthcare monitoring and wearable electronics, sustainably.

## 1. Introduction

In recent years, there has been a significant interest in wearable electronics, which has resulted in the expansion of their engineering applications, such as healthcare monitoring. These technological advancements have made it possible to develop flexible devices that can be worn directly on the skin, allowing for more accurate and continuous monitoring of various health parameters. Moreover, the improvement of flexible and conductive material‐based soft electronics suitable for in vivo organs/tissues offers implantable medical devices, the possibility of electrical stimulation, biochemical sensing, and energy generation in a manner that is both in situ and real‐time [1–3]. For example, Almufleh et al. designed and produced implantable electrodes with exceptional flexibility that can be employed as the ideal transducer, connecting the damaged nerve and the muscle [4]. These electrodes are engineered with a conductive polymer (PANI) and silicone, ensuring optimal control and functionality. Furthermore, mechanical energy harvesters, which have dual purposes to power implantable medical devices and also function as real‐time sensors have been recently developed [5]. This piezoelectric thin film of polarized polyvinylidene fluoride‐based blood pressure monitor was a remarkable innovation in this domain, and it has been evaluated thoroughly for its effectiveness and feasibility, including simulated, in vitro, and in vivo testing. The device demonstrated an excellent linearity between systolic blood pressure and output voltage with high sensitivity. However, those devices utilized in applications that involve direct contact with human tissue must meet strict regulations. In other words, biocompatibility and bioresorbability are essential features of such devices, which should also have the ability to minimize infection, inflammation, and the need for repeat surgeries [6, 7]. On the other hand, developing flexible bioelectronics has been significantly facilitated by synthesizing nanomaterials within a range of sizes and dimensions or modifying their surfaces to meet specific biomedical application requirements [8–11]. Therefore, there is a growing interest in using implantable electronic systems made of biodegradable and biocompatible nanomaterials, which offer a range of potential applications, from diagnostic to therapeutic purposes during physiological processes [2, 12–14]. Combined with their ability to dissolve in the body safely over time, they represent a promising advancement in biomedicine.

The future of self‐powered, biodegradable, and implantable electrical stimulators via nanogeneration incorporating piezoelectric or triboelectric technologies hinges on developing conductive and flexible bioelectrodes [15–17]. However, finding biomaterials that possess both high conductivity and inherent flexibility is a challenge. Nevertheless, numerous strategies have been proposed and implemented to achieve such exceptional properties [18–21]. Among them, the preparation of flexible polymeric films modified with carbon‐based conductive nanomaterials is a simple and effective approach for achieving the desired bioelectrode characteristics. In particular, the process of electrospinning a polymeric solution followed by post‐processing modification yields a remarkable porous structure of a functional hybrid nanofiber network having flexibility and a large surface‐to‐volume ratio [22–24]. This makes it an effective substrate for producing electrodes with suitable electrical conductivity and flexibility. Most recently, a highly efficient method to create a composite electrode made of flexible polymer and carbon nanotubes was reported [25]. A two‐step process, including electrospinning and a scalable ultrasonic cavitation treatment, allowed long carbon nanotubes to be embedded into electrospun polyurethane fibers, creating a dense, mechanically tough, and electrically stable conductive network that surrounds each fiber. The electrodes produced in this way exhibit excellent flexibility, with a recoverable stretching rate of up to 200%. They also have high conductivity, with a low sheet resistance of 30–50 Ω sq^−1^, as well as exceptional stability, withstanding over 20,000 bending and stretching cycles, and are highly durable, sustaining ultrasonic washing for over 30 min. Often, complementary techniques such as 3D printing and patterning are employed to introduce metallic materials on the surface of the fibers to improve their electrical properties while maintaining their flexibility. Recently, Yoon et al. used silver/gold nanowires and core–shell nanoparticles to improve the electrical properties of the fibrous membrane, which improved conductivity and provided sheet resistance as low as 1 Ω sq^−1^. However, a very high ink concentration was used for printing at 24 mg mL^−1^ [26]. Li et al. used a similar strategy proposed in this study for the functionalization of nanofibers by growing metal‐organic frameworks on electrospun nanofibers for use in biofuel cells. Here, they applied sandwiched layers of multiple components such as carbon nanotubes, MOFs, and nanofibers to achieve an open‐circuit voltage of 0.35V and maximum power density of 1.09 W m^3^ at 0.25 V [27]. Similarly, zinc oxide nanorods templated on polypyrrole have been grown on nonwoven nanofiber mat to preserve the flexible character while improving the electrical conductivity of the nanofibers offering an impedance of 15.64 kΩ cm^−2^ [28]. While several more studies have explored the use of conductive carbon‐based additives in flexible substrates [22, 24], none of them have employed bioresorbable and biocompatible nanofibers. Such nanofibers have the potential to encapsulate conductive additives and even be coated with them to enhance the conductivity of the flexible substrate.

In the present study, we have fabricated a new type of bioelectrode by employing electrospun nanofibers from bioresorbable polymer in combination with conducting functional agents such as graphene and PEDOT:PSS. Our focus was on creating a bioresorbable and biocompatible substrate for this purpose. The fabricated nanofibers were characterized using scanning electron microscopy (SEM) to analyze the fibers’ diameter and their surface morphology to ensure the absence of beaded morphology, which can affect the mechanical properties of the fibers. Moreover, the effect of dripping the fibers with conducting functional agents on their morphology was understood. The infrared spectroscopy using ATR‐FTIR was performed to confirm the interactions among functional groups corresponding to the functional agents and polymer matrix, implying their proper incorporation within and on the fibers’ surface [29, 30]. Fibers were also subjected to thermal analyses, both TGA (thermogravimetric analysis) and DSC (differential scanning calorimetry), to measure the thermal stability and analyze the composition compared to the pristine fibers based on their weight changes and residue, whereas the DSC confirmed the changes in thermal transitions, which might have occurred due to the presence of inorganic functional agents affecting the processing of fibers and their application [31, 32]. The mechanical properties of the fibers were further analyzed using tensile testing and their contact angle, which are essential for applications in soft tissue implants. These properties dominate the behavior and interaction of fibers within various components within in vivo and in vitro environments [33, 34]. The conductivity of fiber mats was evaluated to assess their feasibility in electrode applications, after which the most promising electrode material was used in the assembly of the TENG device. Finally, the biocompatibility of the fibers was tested using various standard assays. This work represents a significant development towards the implementation of bioelectrodes for applications such as TENG devices. There it can be used as a replacement for the metal electrode substrate used in current TENG devices. The findings highlight the feasibility and potential of electrospinning electrodes using biodegradable polymers.

In contrast to previously reported biodegradable or polymer‐based electrodes that rely on metallic transient conductors, nonresorbable fillers, or mechanically rigid substrates, the present work introduces a fully polymeric, metal‐free, bioresorbable electrode based on an electrospun PLCL scaffold with dual conductivity enhancement. By combining graphene incorporation within the nanofibers and a conformal PEDOT:PSS coating, we obtain a soft‐tissue‐like, cytocompatible, and electrically functional electrode.

## 2. Experimental Section

### 2.1. Materials

Commercial PLCL (L‐lactide/caprolactone copolymer) (Purasorb PLC 7015) from Purasorb, Corbion, Netherlands, was used as a polymer matrix of electrospun nanofibers. Chloroform and trifluoroacetic acid (TFA) (analytical reagent grade) from Carl Roth, Karlsruhe, Germany, were used as solvents. Poly(3,4‐ethylenedioxythiophene)‐poly(styrenesulfonate) (PEDOT:PSS) as 1.3 wt% dispersion in H_2_O was purchased from Sigma‐Aldrich, Merck KGaA, Darmstadt, Germany. Graphene powder (XT3) and graphene ink (XT8) were provided by Graphene‐XT, Bologna, Italy, and were used without modifications.

### 2.2. Fabrication of Flexible and Conductive Membrane Film by Electrospinning and Drop Coating Technique

Graphene XT3‐blended (4% w/v) polymer solution was prepared by dissolving 23% (w/v) of PLCL (L‐lactide/caprolactone copolymer) in a mixed solvent of TFA/chloroform (3:2, v/v) and magnetically stirring for 10 h. Electrospinning was carried out at 16 kV, a tip‐to‐collector distance of 150 mm, and a solution feed rate of 0.6 mL h^−1^. During electrospinning, the nozzle (23G) moved laterally (i.e., back and forth) on its axis for a distance of 80 mm and a linear speed of 10 mm min^−1^. 4 mL of blended solution was electrospun onto a drum collector connected to −4 kV. After electrospinning, the nanofibrous film was dried at 40°C for 24 h to ensure the removal of the residual solvents. To significantly improve the electrical conductivity, the amount of 2 mL PEDOT:PSS or graphene ink solutions, as obtained from respective manufacturers, were evenly drip coated onto both sides of the circular‐shaped composite nanofibrous membrane (diameter: 40 mm). Next, the membrane was left to dry overnight at room temperature, allowing the solution to fully integrate into the membrane structure. For ease of discussion, the graphene‐incorporated nanofibrous membrane and its coating with PEDOT:PSS or graphene ink will be referred to herein as Gr/PLCL, Gr/PLCL_PEDOT:PSS_, and Gr/PLCL_Gr.ink_, respectively.

### 2.3. Preparation of Polymer Contact Films

Polymer contact layers used to test the suitability of conductive fiber mats as electrodes in TENG devices were prepared from poly(lactic acid) (PLA, Goodfellow ME346310/3) and poly(glycerol sebacate) (PGS). PGS prepolymer was synthesized according to the paper by Timusk et al. [35]. PLA solution was prepared by dissolving 0.2 g of polymer in 2 mL of CHCl_3_, while PGS prepolymer was dissolved in acetone during synthesis to yield a solution with a concentration of approximately 57 wt%. PLA film was drop‐casted into a glass Petri dish to obtain the film that adhered to the fiber mat electrode. The PGS layer on the fiber mat electrode was obtained using sequential spin‐coating and crosslinking in a lab oven at 140°C.

### 2.4. Characterization of Nanofibers

#### 2.4.1. Microscopy, Thermal, and Spectroscopy Analyses

A scanning electron microscope (JSM‐IT 100 InTouchScope) at an accelerating voltage of 20 kV was used to analyze the morphology of electrospun fibers. The fibers were sputtered with a thin coating of gold for SEM using Cressington Sputter Coater 108auto. The resulting micrographs were examined using the ImageJ software, which measured the micrographs at 15 unique locations to provide information about the diameter distribution of the fibers. Statistical analysis was performed using OriginLab.

The nonwoven mat of both pristine and functionalized nanofibers/microfibers was subjected to thermal analysis. DSC and TGA (Perkin Elmer) were used to characterize the thermal properties of electrospun and coated fibers. Using TGA, the effect of immobilization of functionalization agents, such as graphene XT3, as well as coating materials (PEDOT:PSS and graphene ink) on the thermal stability can be studied. Moreover, the weight loss behavior shall confirm the sample composition. Briefly, TGA was performed on cut fiber samples, and ∼10 mg of fibers were taken in a ceramic cuvette, which was subjected to a temperature program running from 35°C to 700°C at a rate of 5°C min^−1^. For performing DSC, approximately 10 mg of fibers were cut and sealed in aluminum pans. These pans along with a blank were analyzed under alternating heating and cooling cycles from −65°C to 230°C at a rate of 5°C min^−1^. The samples were subjected to two heating programs to erase the materials’ thermal processing history, remove residual solvents, and then measure the intrinsic phase change behavior of the composition with the second cycle. Finally, the PerkinElmer Spectrum 2000 spectrometer with the attenuated total reflection (ATR) assembly attached was used to perform Fourier transform infrared spectroscopy (FTIR) analysis of pristine and functionalized fibers at a scanning resolution of 2 cm^−1^. Electrospun fibers were pressed simply against the crystal on the top plate for analysis, and the spectra were recorded.

#### 2.4.2. Tensile Testing of the Fibers

The tensile testing of the nanofibers/microfibers was done using the Tinius Olsen 5ST. A standard method, BS EN ISO 527‐3:1995 for the tensile properties of plastics, films, and sheets, was used for measuring the mechanical properties of the fibers. For this purpose, the fiber sheets were cut into rectangular strips with dimensions 50 × 10 mm. A single representative tensile test run was performed using the gauge length fixed at 20 mm and the tension speed 50 mm min^−1^.

#### 2.4.3. Contact Angle Measurement

The contact angle measurement was performed using a OCA 35 goniometer, with a tilting unit TBU 90E, DataPhysics Instruments, Germany, attached with an iDS UI‐3360CP‐M‐GL R2 camera. The automatic pump assembly was used to drop 5 μL dist. H_2_O onto the samples at various places, and images were recorded. After 10 measurements (*n* = 10/sample) for each sample, the average data were reported.

#### 2.4.4. Conductivity Measurement

Conductivity was determined from resistance measurements on a fiber mat sandwiched between two polished copper electrodes (1 cm × 1 cm). A micrometer was used to determine the thickness of the sample. Resistance was measured using a Keithley 2100 multimeter. Conductivity was calculated using the equation *σ* = *d*/(*A* × *R*), where σ is conductivity, *d* is the thickness of the fiber mat, A is the electrode area, and R is resistance. Resistance was measured in at least five different positions (*n* = 5/sample) of the fiber mat to calculate the average conductivity and standard deviation.

#### 2.4.5. Triboelectric Measurement

The conductive fiber mat was adhered to the glass slide using double‐sided adhesive tape to ensure flat and even support for contact‐separation tests. To compare the fiber mat electrode with the conventional electrodes (Cu and ITO), electrodes were contact‐separated against a commercially obtained PTFE film as a contact layer. To perform triboelectric characterization of chosen polymer materials and the TENG device, conductive fiber mats were covered by polymer contact layers for tests in contact‐separation mode.

The current and voltage generated in the contact‐separation tests were measured under precise conditions: a separation distance of 5 mm, a pressing force of 10 N, and a contact‐separation frequency of 1 Hz. Each cycle consists of a slow approach (≈0.3–0.4 s), a loading phase (≈0.5 s), and a rapid separation (≈0.05–0.1 s). Contact‐separation was carried out using an Instron E1000 material testing machine to ensure repeatability. The generated current signals were measured using a Keithley 6514 electrometer connected to a PicoScope 5444B PC oscilloscope system. Contacts for measurements were mechanically clamped rather than soldered, avoiding thermal effect damages. The measured signal noise in all electrode cases was within instrument baseline and no differences could be observed. Surface charges, *Q* (nC), were calculated from the measured current peaks during separation phase using the equation *Q* = ∫*ⅈ* *·* *ⅆ* *t*, where *i* is the instantaneous current (nA) and *dt* is the differential of time (*s*). Since the instantaneous current *i* depends on the separation speed, the much faster separation phase produces a higher current amplitude on the positive side. Accordingly, charge density was expressed as charge per cm^2^ of contact area between the polymer sample and conductive fiber electrode. Instantaneous power was calculated using *P*(*t*) = *V*(*t*)^2^/*R* from the data obtained in voltage measurements at the corresponding load resistance *R*. Next, the instantaneous power was plotted as a function of time. Integration of the peak area allowed to calculate the energy generated during separation. The peak duration was also determined from the plot and used to calculate the average power during the separation step using *P* = *E*/*t*. Energy density and power density were calculated by dividing energy and power by the sample contact area.

A full wave bridge rectifier was used to convert the AC signal generated by TENG to DC for energy storage. A capacitor with a capacitance of 470 nF was used to store the harvested electrical energy. The voltage across the capacitor was measured using the Keithley 6514 electrometer under identical contact‐separation conditions provided for current and voltage measurements. Energy stored in the capacitor was calculated using *W* = 0.5*C* *V* ^2^, where *C* was the capacitance and *V* voltage across the capacitor plates.

### 2.5. Cytotoxicity/Biocompatibility Studies

The cytotoxicity/biocompatibility studies of the conductive fiber mats were carried out according to an EN ISO 10993‐5:2009 standardized study with the following parameters: human primary dermal neonatal fibroblasts (HDFn) were employed, and the study comprised several methods. Two ways of cytotoxicity/biocompatibility were set up: (A) an extract‐based method where the material is leached in a cell culture medium, which is then added to cultured cells, and (B) direct seeding of cells onto conductive fiber mats. In total, the studies comprised the following endpoints: metabolic activity, proliferation activity for both (A) and (B), cell viability (fluorescent labeling of live and dead cells), and cell morphology (light microscopy, and fluorescent labeling of cytoplasm and nuclei) for (A) only. The endpoints were analyzed 1, 3, and 7 days after the cells were exposed to extracts or seeded onto the fiber mats, respectively.

#### 2.5.1. Cell Culture Conditions

HDFn (LGC Standards, U.S.A.) were cultured using Dulbecco’s Modified Eagle Medium (DMEM; cat. no. D6429, Sigma‐Aldrich) supplemented with 10% fetal bovine serum (FBS; cat. no. 10270106, Gibco), 100 U mL^−1^ penicillin, and 100 μg mL^−1^ streptomycin (cat. no. P4333, Gibco) in a CO_2_ incubator at 37°C (high humidity) with 10% CO_2_ . The cells were passaged once every 3‐4 days. Passage 9 was used for all reported studies.

#### 2.5.2. Cytotoxicity/Biocompatibility Studies: Setup

Small circles (diameter = 6 mm) were cut from the materials using a skin biopsy punch. The materials were then sterilized in ethylene oxide at 37°C and allowed to air out for 1 week at 37°C. In the extract‐based method (A), the materials were leached for 24 h in a complete cell culture medium (250 μL medium/one 6 mm disc) in a CO_2_ incubator at 37°C (high humidity) with 10% CO_2_. 25 mL glass bottles with partially unscrewed caps were used for preparing the extracts. Material‐less medium incubated in the same conditions as the extracts served as a control. Cells were seeded (15,151.5 cells cm^−2^) 24 h before the addition of the extract in a 96‐well plate in 200 μL of the complete cell culture medium. 24 h after cell seeding, 200 μL of extract or control medium was added to the cells and incubated for 1, 3, and 7 days. Direct cell seeding onto conductive fiber mats (B) was carried out as follows: small circles (diameter = 6 mm) were punched out of the fiber mats, sterilized as described previously, and placed in a 96‐well plate. Then, they were prewetted using 20 μL of complete medium/circle. The cells were seeded directly onto fiber mats (7500 cells/circle) in 50 μL medium/circle. Seeded cells were allowed to attach to the scaffolds for 1.5 h in an incubator (at 37°C (high humidity) with 10% CO_2_) and the medium was filled up to 200 μL afterward. Cells seeded on tissue culture polystyrene were used as a control (untreated cells) in both experiments. For both direct and indirect in vitro cell culture, two replicates and two pictures per sample were analyzed.

#### 2.5.3. Cell Metabolic and Proliferation Activity

1, 3, and 7 days after the extracts were added to the cells (A) or after the cells were seeded onto fiber mats (B), the metabolic activity of cells was measured via MTS assay (CellTiter 96 AQueous One Solution Cell Proliferation Assay; Promega, Madison, WI, USA) according to the manufacturer’s instructions. Mitochondria of viable cells are capable of converting the assay’s substrate into a soluble formazan absorbing light at 490 nm. The readings were performed via a microplate reader (Infinite M200 PRO, Tecan, Switzerland). After the reading, a lysis buffer was added to the samples, and the total amount of double‐stranded DNA (dsDNA) in the cell lysate was measured using the Quant‐iT dsDNA Assay Kit High Sensitivity (Life Technologies, Carlsbad, CA, USA) according to the manufacturer’s instructions. Six replicates were used for both metabolic activity and proliferation testing.

#### 2.5.4. Cell Morphology and Viability

Cell morphology was examined by light microscopy using Olympus IX70 with an Olympus DP80 camera and a 20x objective. The morphology of cells was also analyzed via fluorescent staining of cells’ components: fluorescent probe 3,3′‐diethyloxacarbocyanine iodide (DiOC6(3); Invitrogen, USA) staining the cytoplasm and propidium iodide (PI; Merck, USA) staining the nuclei were used for the purpose. Briefly, the cells were rinsed with phosphate‐buffered saline (PBS) and fixed with 2% paraformaldehyde in PBS for 10 min. After fixation, the cells were rinsed with PBS and permeabilized with 0.1% Triton X‐100 in PBS for 15 min. Following permeabilization, the cells were rinsed with PBS and stained with 10 μg mL^−1^ of DiOC6(3) in PBS for 1 h. The cells were rinsed again with PBS and stained with 5 μg mL^−1^ of propidium iodide in PBS for 10 min. Lastly, the cells were rinsed with PBS and visualized using a fluorescence microscope (Olympus IX70 with an Olympus DP80 camera); DiOC6(3): *λ*_ex_/*λ*_em_ = 488/505–550 nm; PI: *λ*_ex_/*λ*_em_ = 561/630–700 nm.

Cell viability was analyzed using fluorescent live/dead staining. To stain living cells, 10 μM of 2′,7′‐bis(2‐carboxyethyl)‐5(6)‐carboxyfluorescein acetoxymethyl ester (BCECF‐AM; Sigma‐Aldrich, Saint Louis, MO, USA) probe was added to the cells in complete medium and the cells were incubated at 37°C for 30 min while protected from light. The cells were then washed with PBS and incubated with PI (5 μg mL^−1^) for 10 min at 37°C while protected from light. PI does not penetrate the membrane of viable cells, thus staining the nuclei of dead cells only. After PI incubation, the cells were rinsed with a complete medium and visualized via Olympus DP80 with a 10x objective. BCECF: *λ*_ex_/*λ*_em_ = 503/528 nm; PI: *λ*_ex_/*λ*_em_ = 561/630–700 nm. Here, two replicates and two pictures per sample for cell morphology and cell viability staining.

### 2.6. Statistical Analysis

Statistical analysis was performed using GraphPad Prism 8. Normality was tested by the Shapiro–Wilk test for each experiment and each experimental time point. One‐way ANOVA with Tukey’s multiple comparisons test was then performed to compare the treated experimental groups with the untreated cells (control). Statistical differences (*p* value ≤ 0.05) between experimental groups and untreated cells are marked via straight lines, and asterisks connect the respective compared experimental groups to the untreated cells.

## 3. Results

Fabrication of a bioelectrode that was functionalized with graphene XT3 and PLCL polymer as the matrix by simple electrospinning resulted in a flexible nonwoven nanofiber mat with approx. dimensions 10 × 15 cm. The nanofibers were further processed by coating them with commercially available conducting polymer PEDOT:PSS and graphene ink. The functionality, physicochemical properties, and biological behavior of developed compositions were tested via several standard analyses.

### 3.1. Morphological Analysis

A scanning electron microscope was employed to identify the underlying morphology of the fabricated material, the influence of the inclusion of graphene, and the effect of coating on the surface of the fibers. As seen in Figure 1(a), the pristine nanofibers appear smooth with the absence of the beads, very similar to the nanofibers obtained from the solution containing graphene XT3 blended with PLCL polymer (Figure 1(b)). The average diameters of the nanofibers/microfibers as measured with ImageJ were 217 ± 87 nm, 273 ± 127 nm, 1143 ± 450 nm, and 1228 ± 446 nm, for PLCL, Gr/PLCL, Gr/PLCL_PEDOT:PSS_, and Gr/PLCL_Gr.ink._, respectively. As expected, the nanofibers obtained directly after electrospinning possessed the lowest diameter, and graphene‐blended PLCL nanofibers showed an increase in average diameter. The fibers coated with graphene ink or PEDOT:PSS showed an average diameter significantly higher than preprocessed dimensions, largely due to the coating layer from dripping the fibers with functional agent solutions (Figures 1(c) and 1(d)). The fibers showed some swelling during the coating process, and as seen in SEM micrographs, the fibers had a larger deviation in their diameters after drying. The fibers that were coated with PEDOT:PSS appeared flatter in their morphology as compared to the graphene ink‐coated fibers when observed under SEM.

FIGURE 1SEM micrograph of pristine PLCL copolymer, which was electrospun as the matrix for bioelectrode (a); PLCL nanofibers functionalized with graphene (Gr/PLCL) (b); PLCL nanofibers functionalized with graphene and coated with PEDOT:PSS (c); PLCL nanofibers functionalized with graphene and coated with graphene ink (d).

**Figure:**
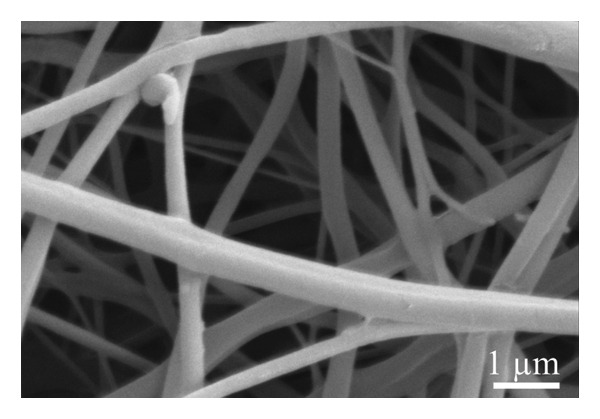
(a)

**Figure:**
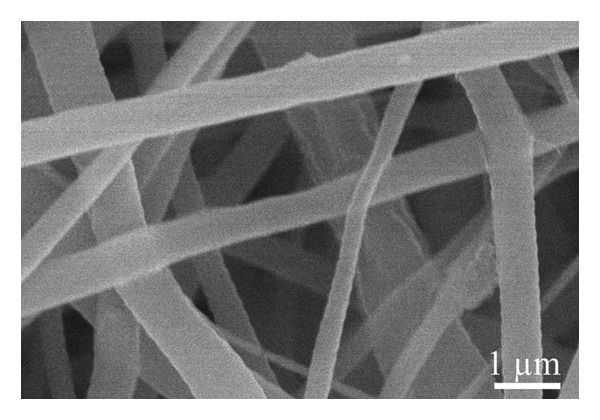
(b)

**Figure:**
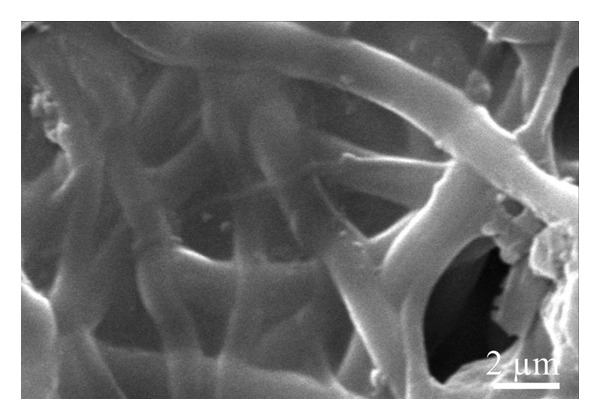
(c)

**Figure:**
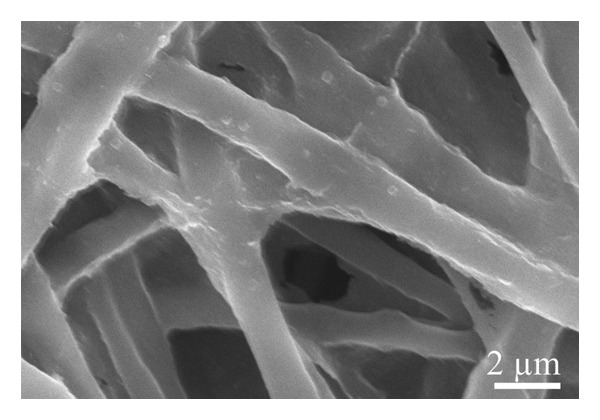
(d)

The contact angles of the prepared samples were measured to determine the intrinsic water wettability of graphene‐functionalized PLCL copolymer electrospun as a base for electrode materials and compared them to pristine nanofibers. Moreover, the effects of the different conductive coatings (PEDOT:PSS and graphene ink) on their surface hydrophobicity were also investigated. As the application of fibers requires being surrounded by a biological medium, where there are expected interactions with proteins and other biomolecules, establishing the surface properties of the electrodes will help a great deal in predicting their effectiveness and stability [36–38]. Moreover, the surface properties of materials can influence the adhesion properties, which in turn affect the generation of triboelectric charge [39]. As shown in Figure 2, the pristine and graphene‐functionalized nanofibers exhibit hydrophobic surfaces with average contact angles of 122.36 ± 4.87° and 124.4 ± 8.49°, respectively. Furthermore, we observed a remarkable superhydrophilicity phenomenon when graphene ink was used as a conductive coating layer on the graphene‐functionalized nanofibers. The hydrophilic nature of the graphene ink on the coated surface caused the water droplet to vanish instantly, followed by a swift and complete spread of the droplet on the functionalized membrane, indicating full wetting, whereas the Gr/PLCL_PEDOT:PSS_ fibers showed a contact angle of 82.5 ± 10°. Coating of the nanofibers with PEDOT:PSS decreased the contact angle as expected due to its hydrophilic nature [40].

FIGURE 2Contact angles with (a) photographs and (b) the bar chart of contact angles’ measurements of all samples.

**Figure:**
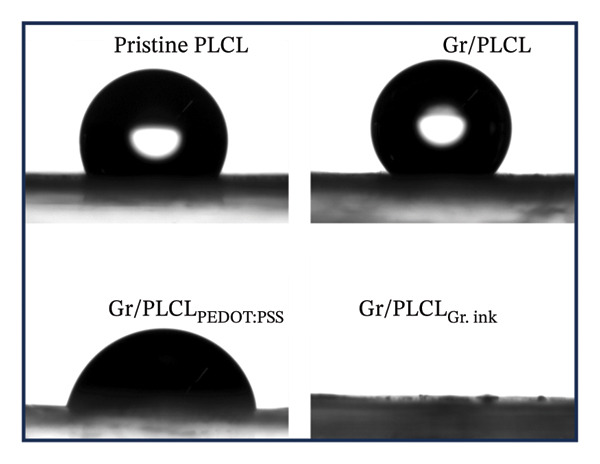
(a)

**Figure:**
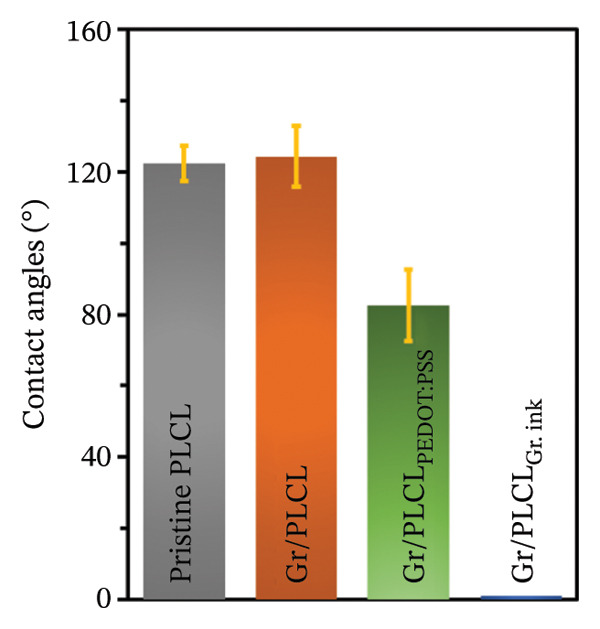
(b)

### 3.2. Tensile Testing

The tensile testing of the nanofibers before and after coating. The Young’s modulus was calculated based on the results (Supporting Information Figures S1 and S2). It was observed that the sample Gr/PLCL_PEDOT:PSS_ possessed the highest Young’s modulus at 98.8 ± 15.8 MPa, followed by PLCL 49.3 ± 2.1 MPa, Gr/PLCL 46.6 ± 4.6 MPa, and Gr/PLCL_Gr.ink_ 33.7 ± 2.0 MPa. This shows that while there is some change in the Young’s modulus values after graphene functionalization, it is not significantly different than the pristine nanofibers. The tensile values obtained for the fibers closely match the mechanical properties of the soft tissues present in the body [41, 42]. Such mechanical properties should make the bioelectrode conform without inducing mechanical stresses. The Gr/PLCL_PEDOT:PSS_ sample’s remarkably significant modulus was evident during the coating procedure itself, as it rendered a glossy film over the fiber mat, and the texture was changed. The presence of PEDOT:PSS on the surface of the fiber mat, creating a film, is evident from Figure 1(c). Therefore, it has much better intermaterial interaction, which is not the case in flaky graphene oxide, and the flakes easily disassociate from each other. Overall, the tensile test reveals that Young’s modulus lies within the range of the soft tissues, which should provide the necessary conformity and properties to the bioelectrodes for use in implants [43, 44].

### 3.3. TGA

The study utilized TGA to analyze the impact of functionalization with graphene XT3, as well as conductive coating materials (PEDOT:PSS and graphene ink), on the thermal stability of PLCL copolymer electrospun fibers, moreover, to confirm the composition of the samples via weight loss behavior. The results are presented comprehensively in Figure S4 as TGA and differential thermogravimetric (DTG) curves. The degradation of the pristine PLCL fiber membranes commenced at around ∼265°C and concluded at about ∼420°C with almost complete weight loss [45]. The PLCL polymer degraded in a single step, however, when graphene‐functionalized nanofibers Gr/PLCL were analyzed, they showed a two‐step degradation behavior. The two‐step degradation of the fibers can be confirmed via the DTG curve in Figure S4b. The first step occurred within the ∼239°C–365°C temperature range, resulting in the amorphous PLA decomposition. The second step occurred within the temperature range of 365°C–420°C, which usually leads to the decomposition of the low‐crystallinity polycaprolactone. However, it is to be noted that this is the same temperature at which the graphene oxide has been shown to be degraded via the breaking of the carbon backbone into volatile gases such as CO [46, 47]. Hence, the two‐step degradation is prominent in the samples containing higher amounts of graphene as part of the total composition. Further, it has been observed that the temperature at which nanofibers begin to degrade was slightly altered when coated or functionalized with graphene compared to when they are in their pristine form. One possible reason for the faster degradation of these nanofibers is the loss of acidic functional groups and residues during the exfoliation process used to prepare graphene [48]. Nevertheless, by immobilizing the graphene in PLCL, a decrease in weight loss was observed compared to the pristine material. Additionally, the electrically conductive PEDOT:PSS polymer coating on the nanofiber membrane has led to an increase in the thermal decomposition temperature. The DTG analysis of the Gr/PLCL and Gr/PLCL_Gr.ink_ nanofibers revealed two distinct peaks corresponding to their maximum degradation temperature. On the other hand, the PLCL and Gr/PLCL_PEDOT:PSS_ samples exhibited only one decomposition process, with the main peak at ∼333°C and ∼378°C, respectively. The temperatures at major weight loss events are listed in detail in Table S1. In general, the high chain compactness that arises from the interaction between PLCL chains and graphene is believed to significantly enhance the T_Endpoint_ of hybridized nanofibers, such as Gr/PLCL, Gr/PLCL_PEDOT:PSS_, and Gr/PLCL_Gr.ink_. After TGA, it was evident that coating the fibers with PEDOT:PSS has a significant influence on the mass degradation behavior of fibers. The presence of the PEDOT:PSS on the surface of PLCL prevented the formation of char at higher temperatures [49]. Overall, the weight loss measurement has shown that the coatings with Gr.ink and PEDOT:PSS lead to 89% and 84% material loss, compared to 97.6% for pristine samples.

### 3.4. DSC

To characterize the fabricated materials and analyze the changes in thermal transitions, which might have occurred due to the presence of inorganic functional agents, DSC was performed along with TGA. The samples were measured using a typical temperature program, increasing the temperature from −65°C to 220°C in the first heating scan. The sample was cooled using the same rate to −65°C and heated again to 230°C to record the change in the thermal behavior of the samples. As seen in Figure S5a, the first heating scan in DSC measurement shows that the nanofibers exhibit glass transition temperatures (*T*_*g*_) of 39°C, 44°C, 45°C, and 41°C for PLCL, Gr/PLCL, Gr/PLCL_PEDOT:PSS_, and Gr/PLCL_Gr.ink_, respectively. The addition of functional agents, either by blending or by coating, resulted in increased *T*_*g*_ values. The sample coated with PEDOT:PSS showed the highest increase in *T*_*g*_. The same trend could be seen repeating in the second heating cycle, where the samples were heated again after controlled cooling (Figure S5b). Further, heating the samples shows an endothermic peak, corresponding to the melting of the samples, with the values noted in Table S2. Interestingly, the second heating cycle shows a slight increase in *T*_*g*_ for all the samples. This points out the changes in the degree of macromolecular ordering, which induced imperfections in PLCL crystals and an increase in the amorphosity of the sample [50]. This phenomenon is confirmed by the thermal behavior of the samples with increasing temperature as the polymer chain relaxes and the melting phase change (*T*_*m*_) occurs (Figure S5b). Here, the effect of reduced crystallinity can be observed via a slight decrease in the melting endotherm temperature peak [51, 52]. The electrospinning process has yielded mainly amorphous PLCL nanofibrous membranes, which aligns with recent reports on the same copolymer matrix [53].

### 3.5. Attenuated Total Reflection‐Fourier Transform Infrared (ATR‐FTIR) Spectroscopy

**FIGURE 3:**
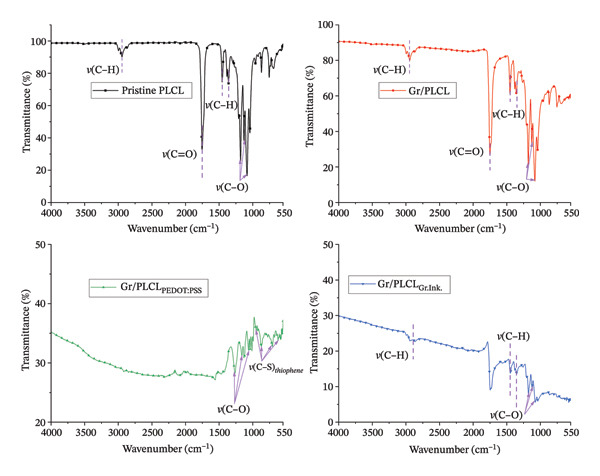
ATR‐FTIR spectra for pristine PLCL nanofibers compared to the nanofibers functionalized by blending with graphene XT3 (Gr/PLCL) and functionalized nanofibers coated with PEDOT:PSS (Gr/PLCL_PEDOT:PSS_) and graphene ink (Gr/PLCL_Gr.Ink_).

To confirm the presence of various functional groups and the effect of coating the fibers with various conducting functional agents, ATR‐FTIR analysis was performed. Here, functionalized and coated nanofibers were compared to the pristine PLCL nanofibers. Transmittance spectra, as seen in Figure 3, were obtained from the analysis. It can be noticed directly that the transmittance by the fibers coated with conducting materials is significantly lower than that of the pristine fibers or Gr/PLCL. Notably, the pristine nanofibers, Gr/PLCL Gr/PLCL_Gr.ink_, showed a methyl (‐CH_3_) stretching vibration absorption peak around 2950 cm^−1^ [54], which was absent in sample Gr/PLCL_PEDOT:PSS_. A strong ester (‐C=O) absorption band can be observed for the samples PLCL, Gr/PLCL, and Gr/PLCL_Gr.ink_ between 1750 and 1725 cm^−1^. The aforementioned absorption bands associated with the presence of PLCL polymer are absent for the samples coated with PEDOT:PSS. Further, these absorption vibrations are subtle in the case of coating with graphene ink. Typical absorption bands associated with symmetric and asymmetric ‐CH_3_ bending vibrations in PLCL can be observed for samples PLCL, Gr/PLCL, and Gr/PLCL_Gr.ink_ at 1450 cm^−1^ and 1360 cm^−1^ [45, 54]. Furthermore, several peaks in the region 1250–1050 cm^−1^ were assigned to the C‐O‐C stretching and were seen in the case of all the samples with varying degrees of strength. The transmittance spectra displayed by Gr/PLCL_Gr.ink_ fibers resemble more or less the characteristic transmittance bands of the graphene [55]. Finally, in the spectrum of Gr/PLCL_PEDOT:PSS_, the bands at 870 cm^−1^, 707 cm^−1^, 627 cm^−1^, and 926–946 cm^−1^ corresponding to thiophene ring ‐C‐S‐ stretch in PEDOT are visible [56].

### 3.6. Triboelectric Measurements

In order to assemble the bioresorbable TENG device, the electrical conductivity of the three PLCL fiber mats functionalized with the graphene XT3 was evaluated. Conductivity was calculated from the resistance measurements considering the dimensions of fiber mats. Accordingly, the highest conductivity was obtained for Gr/PLCL_PEDOT:PSS_ fiber mat; the average value reached 0.65 ± 0.27 mS m^−1^ (Figure 4(a)). In the case of the Gr/PLCL_Gr.ink_ fiber mat, conductivity was lower at 0.03 ± 0.02 mS m^−1^ on average. Generally, materials used as electrodes have electrical conductivity in the range of 10^3^–10^6^ S m^−1^ (copper, aluminum, and platinum) to ensure efficient electron transfer. However, these materials often allow neither flexibility nor compatibility with the human tissue required for bioresorbable devices. Additionally, the requirements for electrode conductivity depend on the intended application and device. Nevertheless, both coated fiber mats show a substantial increase in electrical conductivity compared to uncoated Gr/PLCL fiber mats, for which conductivity was below nS m^−1^, around 1 × 10^−10^ S m^−1^. Further advances in coating technology could improve the conductivity of the coated fiber mats.

FIGURE 4(a) Conductivity comparison of electrospun Gr/PLCL fiber mats and (b) comparison of the generated short‐circuit current for Gr/PLCL_PEDOT:PSS_, Cu, and ITO electrodes in contact with PTFE.

**Figure:**
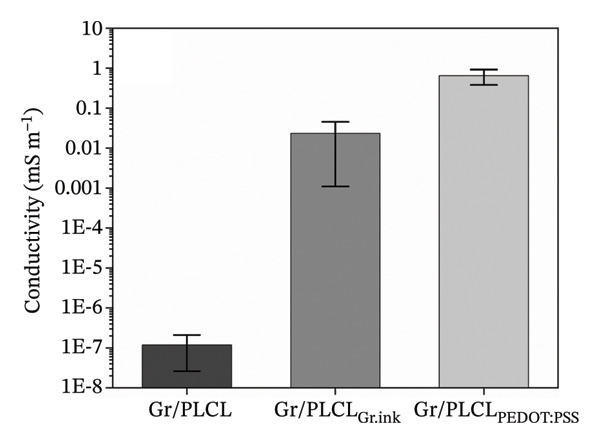
(a)

**Figure:**
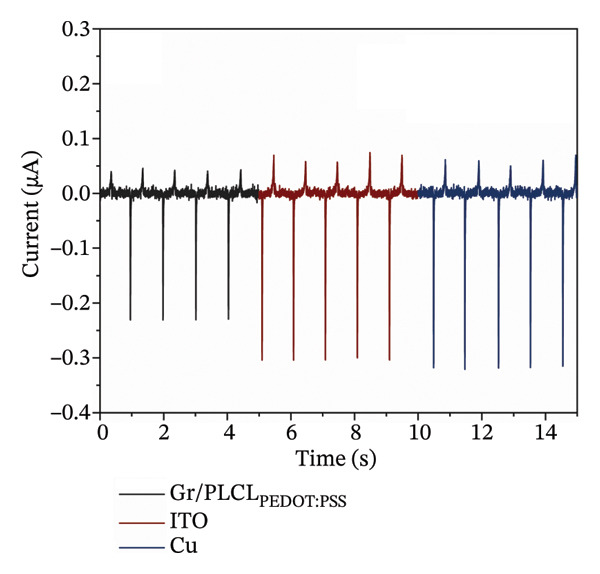
(b)

To ensure that the achieved conductivity is suitable for use in TENG devices, the Gr/PLCL_PEDOT:PSS_ fiber mat was tested as an electrode against one of the most extensively used polymers in TENGs, PTFE, and its performance was compared with that of common TENG electrodes such as ITO (indium tin oxide) and Cu foil under identical testing conditions. Short‐circuit current measurements against the ground show that, after contact with Gr/PLCL_PEDOT:PSS_, the current reached approximately 0.230 μA, corresponding to a charge density of −0.615 ± 0.004 nC cm^−2^ (Figure 4(b)). Since ITO and Cu exhibit conductivities that are three to six orders of magnitude higher, the average currents measured for PTFE in contact with these electrodes were slightly higher, reaching 0.305 μA and 0.316 μA, respectively. The corresponding charge densities were −0.834 ± 0.006 nC cm^−2^ for ITO and −0.820 ± 0.007 nC cm^−2^ for Cu. These results indicate that higher electrode conductivity primarily facilitates more efficient charge transport and current extraction in the external circuit, rather than directly increasing the amount of triboelectric charge generated at the interface. The observed differences in charge density may also be partially attributed to a reduced effective contact area between the fibrous Gr/PLCL_PEDOT:PSS_ mat and the PTFE film, compared to the relatively flat surfaces of ITO and Cu electrodes. In practical TENG devices, both electrodes are typically covered by polymer layers; therefore, the fibrous surface morphology is not expected to adversely affect device performance. Overall, these results demonstrate that Gr/PLCL_PEDOT:PSS_ meets the conductivity requirements for reliable operation as a flexible TENG electrode.

Evidently, Gr/PLCL_PEDOT:PSS_ was chosen as an electrode to create the triboelectric contact layers. Regarding the polymer on contact layers, two biocompatible polymers were chosen, commercial PLA and PGS, previously successfully synthesized by our group [35]. These polymers were also chosen due to their different mechanical properties; PLA is relatively rigid compared to elastomeric PGS. Our previous works have shown that for efficient charge generation and, thus, energy harvesting, asymmetric mechanical properties of contact layers are crucial [57, 58]. Both contact layers were characterized by current measurement against the ground to determine the charge polarity and magnitude as shown in Figure 5. Results revealed that PLA charges positively, with a charge density of +0.169 ± 0.002 nC cm^−2^, while PGS charges negatively, with a charge density of −0.183 ± 0.003 nC cm^−2^. This is in line with previous observations that the stiffer polymer from the pair tends to acquire a positive charge while the more elastic counterpart charges negatively [59].

FIGURE 5Measurement schematic and short‐circuit current of (a) PLA contact layer after contact with PGS and (b) PGS contact layer after contact with PLA.

**Figure:**
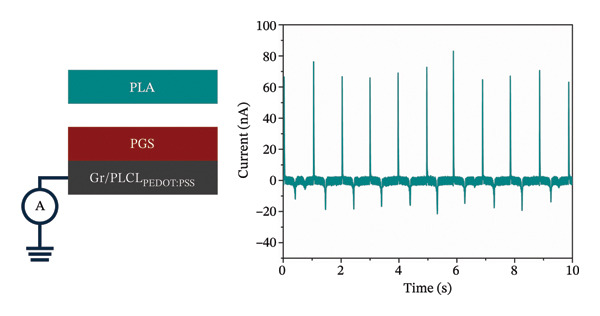
(a)

**Figure:**
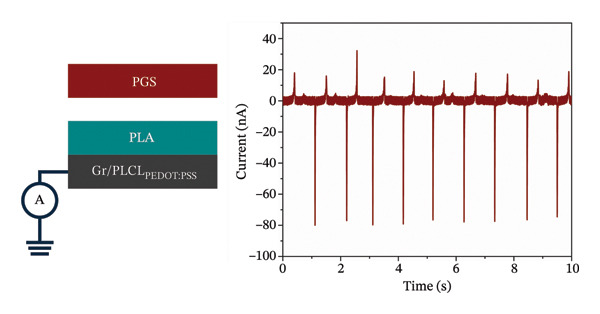
(b)

Next, both contact layers were assembled in a TENG device as shown in Figure 6(a). Short‐circuit current measurement showed that the current reached 0.379 ± 0.034 μA with the calculated charge density of 0.417 ± 0.015 nC cm^−2^ (Figure 6(b)). Open‐circuit voltage was measured at different load resistance values, with the highest voltage of 51 ± 1 V at 1 GΩ resistance (Supporting Information Figure S3). The measured voltages were used to calculate energy and power densities (Figure 6(c)). Voltage as a function of load resistance is shown in Supporting Figure S6. Similarly, as with open‐circuit voltage, the highest energy of 138.95 ± 4.77 μJ m^−2^ was obtained at a load resistance of 1 GΩ. However, the power density reached the maximum value of 1939 ± 19 μW m^−2^ at 60 MΩ resistance, thus indicating that the internal resistance of the TENG device is close to this optimal load resistance value. In context, studies on biodegradable and bioresorbable TENGs cover a wide range of power densities. Biodegradable systems based on cellulose, PLA, or biopolymer composites can reach several hundred mW m^−2^ [60–62]. However, bioresorbable TENGs designed for implantable applications typically operate in a lower power density window, 0.1–100 mW m^−2^ [63, 64]. These devices often use metal electrodes (Mg and Zn) or highly conductive inorganic fillers to boost output [14]. In comparison, the maximum power density of our TENG device (1.9 mW m^−2^) falls in the lower part of this range, however, it is achieved with a polymeric, metal‐free, bioresorbable electrode that also matches the stiffness of soft tissue. The relatively modest output is mainly due to incomplete percolation of conductive pathways in the porous nanofiber mat (evidenced by conductivity studies) and the low‐frequency (1 Hz) contact‐separation. It is well established in triboelectric studies that higher contact force and faster separation increase the displacement current and electrical power output. Also, direct comparison of power density among different biodegradable TENGs must be made cautiously, as methodologies vary across the literature: some studies report average power per cycle (as done here), while others calculate power from the maximum instantaneous voltage. For example, the use of the maximum voltage value would increase our reported power density by an order of magnitude (around 15 mW m^−2^).

FIGURE 6(a) Schematic of a TENG device based on PGS and PLA contact layers with Gr/PLCL_PEDOT:PSS_ as electrodes; (b) short‐circuit current measurement of TENG; (c) energy and power densities of TENG at different load resistance values; (d) schematic of capacitor charging using TENG and (e) change in the voltage of the capacitor during charging for 1 min.

**Figure:**
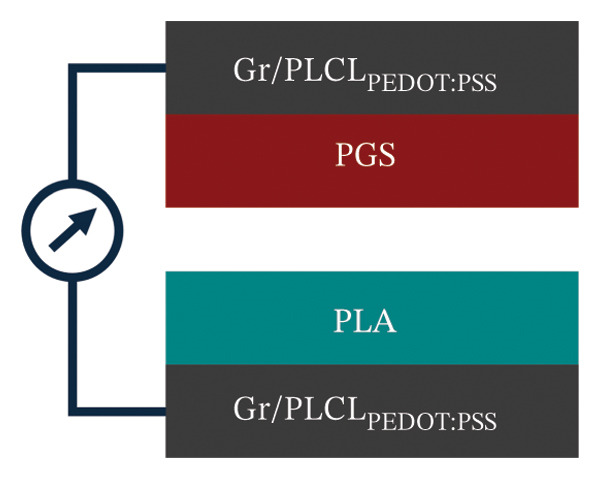
(a)

**Figure:**
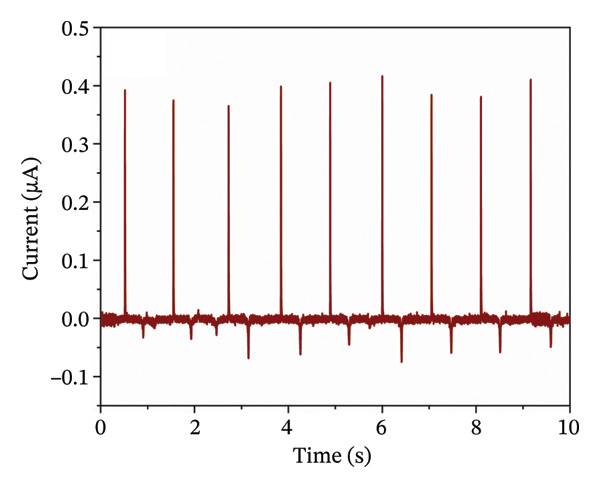
(b)

**Figure:**
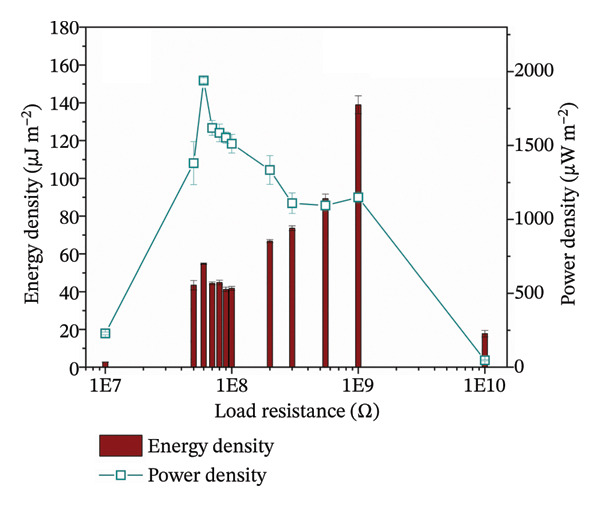
(c)

**Figure:**
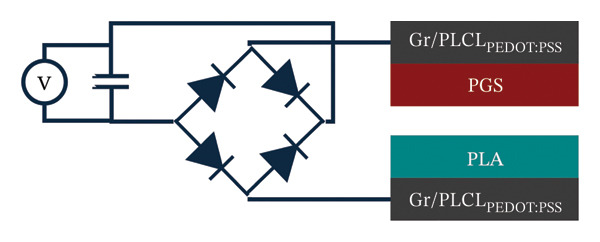
(d)

**Figure:**
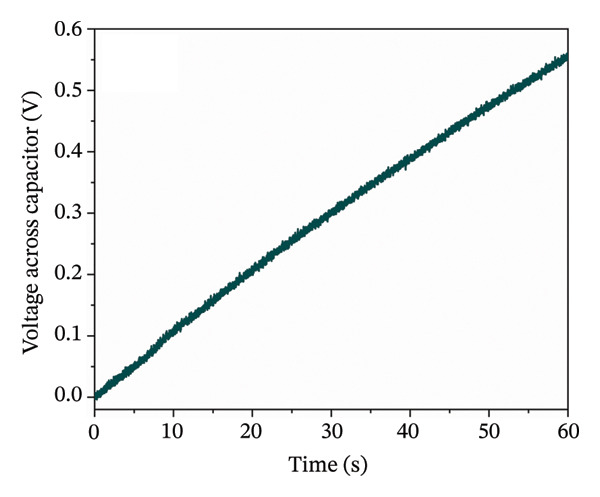
(e)

A full wave bridge rectifier was connected to the TENG device to achieve a more practical demonstration of energy harvesting, as shown in Figure 6(d). The rectified DC signal was used to charge a capacitor with a capacitance of 470 nF. The TENG output signal was not additionally corrected; during charging, we monitored only the voltage across the capacitor, not the TENG itself. The capacitance of 470 nF was chosen because it provides a measurable and reproducible voltage rise within ∼1 min of charging under our test conditions. As is well known, there is a trade‐off between capacitance C and the amount of energy that can be usefully stored in a given charging time: lower‐C capacitors tend to reach higher voltages (and in our configuration, often higher stored energy), but they deliver very small discharge currents, whereas higher‐C capacitors charge to lower voltages but can supply larger currents that are closer to practical load requirements. At the same time, higher‐C capacitors typically exhibit higher leakage currents, which can limit the net energy stored over a fixed time window.

During 1 min of charging from the 5 cm^2^ contact area, the TENG device charged the capacitor to 0.55 V, corresponding to the energy of approximately 71 nJ (Figure 6(e)). Increasing the size of the TENG device and thus increasing the harvested energy could lead to potential use in pulsed electrical stimulation of bone growth or other biomedical applications [65]. The subsequent discharge behavior was analyzed both with external resistors and under open‐circuit conditions. While RC time constants with different load resistances follow the expected *τ* = RC relationship, a more interesting parameter for practical TENG energy storage is the self‐discharge constant (Supporting Figure S7 and Table S3). Under open‐circuit conditions, the capacitor exhibited a self‐discharge time constant of approximately 10^4^ s, corresponding to an effective leakage resistance in the tens of G Ω range. This long relaxation time indicates very low intrinsic leakage and demonstrates that the harvested charge can be retained for extended periods, whereas the RC time constants with external loads describe how rapidly different load resistances would extract that stored energy.

Upon implantation, the TENG device would be subjected to higher humidity than during the lab tests (around 35% RH), which could significantly contribute to surface charge dissipation, thus greatly reducing the output of the TENG device. High humidity has been extensively studied as a contributor to lower TENG performance; therefore, different routes have been proposed to circumvent this disadvantage. One of the surest ways to protect the device is to use encapsulation. Encapsulation mitigates and delays the effects of humidity on the device’s performance. Notably, silicone‐based encapsulants have been widely reported to meet the biocompatibility and barrier requirements necessary for implantable applications, thus supporting the long‐term stability and functionality of the device under physiological conditions [66].

### 3.7. Cytotoxicity/Biocompatibility Studies

Metabolic activity (Figure 7(a)) and dsDNA content as an indication of cell metabolic and proliferation activity (Figure 7(b)) of HDFn cells cultured in the presence of conductive fiber mats’ extracts were analyzed. All three types of functionalized nanofiber samples showed outstanding in vitro biocompatibility, with none of them leading to a decrease in metabolic viability below 70% of untreated control cells (red horizontal line in Figure 7(a)) cultured on tissue culture plastic (EN ISO 10993‐5:2009) on any of the experimental days. On the contrary, there was an increased metabolic activity following 1‐day incubation with extracts of all tested materials and for Gr/PLCL and Gr/PLCL_PEDOT:PSS_, following a 3‐day incubation (Figure 7(a)). An increase in metabolic activity following exposure to xenobiotics signifies either a stimulatory or protective effect [67]. In this case, the proliferation data suggest the former due to all tested materials exhibiting an increase in quantified DNA from day 1–3. The increase in metabolic activity is a phenomenon described previously when cells are exposed to xenobiotics, and unless coupled with a drastic decrease in cells’ activity, it would not raise any concern [68–70]. Moreover, graphene‐containing scaffolds were reported to have elevated cell metabolic activity in studies pertaining to nerve regeneration [71, 72], bone tissue engineering/regeneration [73–75], cardiac tissue regeneration [76], and similar to our experimental setting, human dermal fibroblasts [77].

FIGURE 7Metabolic activity (a) and proliferation activity (b) of HDFn cells incubated in the presence of conductive fiber mats’ extracts 1 (D1), 3 (D3), and 7 (D7) days after the extracts were added to the culture. The dashed line represents the 100% viability of untreated cells, and the solid red line represents the cytotoxic threshold of 70% viability of untreated cells. The results were compared to untreated cells using one‐way ANOVA with Tukey post hoc test; ^∗^ *p* ≤ 0.05, ^∗∗^ *p* ≤ 0.01, ^∗∗∗^ *p* ≤ 0.001, and ^∗∗∗∗^ *p* ≤ 0.0001.

**Figure:**
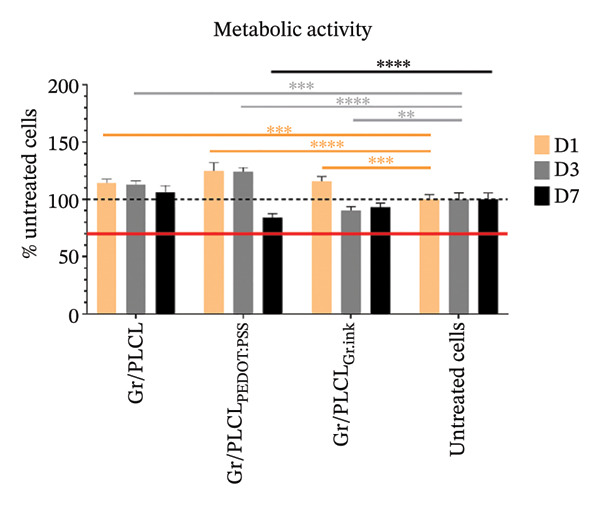
(a)

**Figure:**
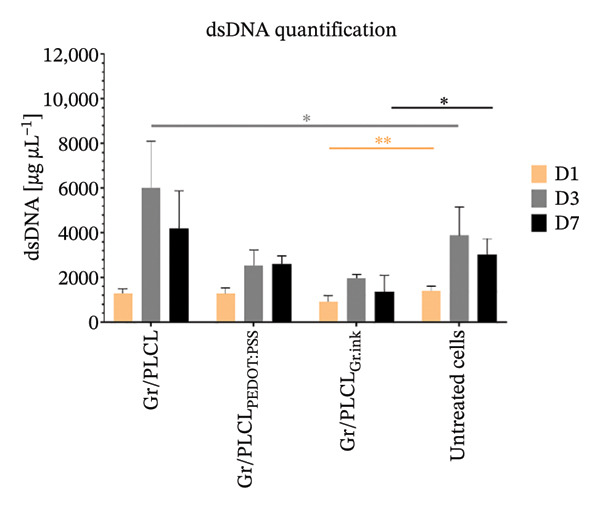
(b)

As already stated, Gr/PLCL extract led to an increase in cell metabolic activity compared to the control cells on Days 1 and 3 after the extracts were added. The quantified DNA is in accordance with this, exhibiting a significant increase in proliferation rate on Day 3 (Figure 7(b)). Similar to Gr/PLCL, the Gr/PLCL_PEDOT:PSS_ extract induced an increase in metabolic activity on Days 1 and 3 after extract addition; however, a lowered metabolic activity on Day 7 (84.43 ± 3.47%) was observed. The decreased metabolic activity on Day 7 nonetheless did not exceed the cytotoxic threshold of 70% viability of control cells. When comparing metabolic activity with proliferation activity, where Days 3 and 7 exhibited comparable values of proliferation rate, one concludes that the Gr/PLCL_PEDOT:PSS_ copolymer stimulated metabolic activity during the first 3 days, followed by a stagnation leading to comparable values of quantified DNA on Days 3 and 7. PEDOT:PSS has already been reported to induce cytotoxicity towards murine RAW 264.7 macrophages in high doses after 24 h [78]. A study in which cardiomyoblasts were exposed to low PEDOT:PSS concentrations in a gelatin‐based scaffold showed elevated metabolic activity [79]. In another study, this time using endothelial cells, there was no cytotoxic or stimulatory effect observed when the cells were exposed to different PEDOT:PSS concentrations as a coating of gelatin/alginate‐based scaffold [80]. Biocompatibility of PEDOT:PSS seems to depend on its dose and administration manner [81]. In our case, even a 7‐day long exposure did not lead to a critically lowered cell viability.

Lastly, the extract of Gr/PLCL_Gr.ink_ led to an increase in metabolic activity on Day 1, followed by a decrease on Days 3 and 7. Again, though significant, the observed decrease did not lead to viability lower than 70% of control cells. Some studies have demonstrated the cytotoxic effect of graphene‐based nanomaterials towards human endothelial cells [82, 83] and rat ventricular cardiomyocytes (interestingly for those materials where the mass fraction of graphene was lower than 0.5%) [84]. As opposed to the former, we have observed a lowered metabolic activity of Gr/PLCL_PEDOT:PSS_ (Day 7) and Gr/PLCL_Gr.ink_ (Day 3) while metabolic activity for Gr/PLCL remained higher/comparable to the untreated control. This would lead one to the conclusion that both decreases in cell viability were caused by the coating and not the copolymer itself. Cytotoxic effects of nanomaterials containing graphene are dependent on an array of physicochemical properties, as well as the matrix in which graphene is embedded [73, 77, 83–90]. Nevertheless, our findings that none of the materials lead to a cytotoxic effect agree with those of Fernández‐Pampín et al. [87], where pristine graphene caused no irritation on a reconstructed human skin model.

**FIGURE 8:**
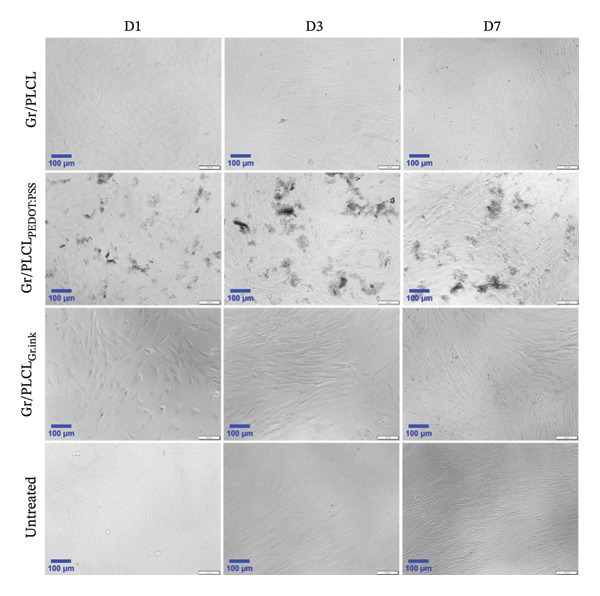
Morphology of HDFn cells cultured either in standard conditions (untreated) or incubated with conductive fiber mats’ extracts on Days 1 (D1), 3 (D3), and 7 (D7), visualized using light microscopy, scale bar ∼100 μm.

**FIGURE 9:**
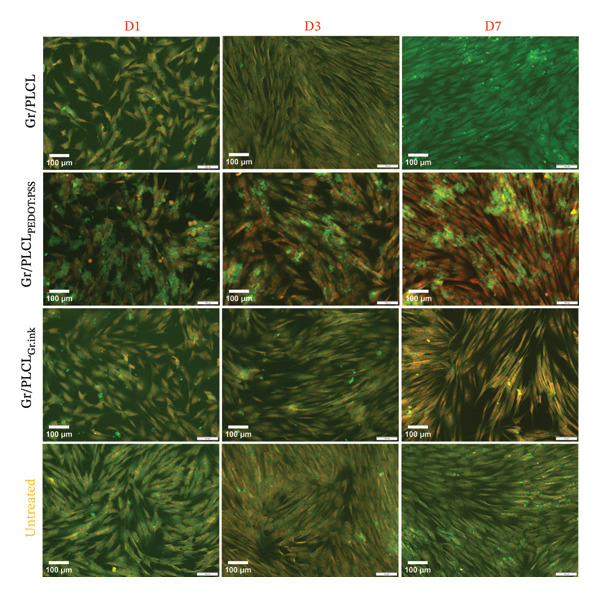
Morphology of HDFn cells cultured either in standard conditions (untreated) or incubated with conductive fiber mats’ extracts on Days 1 (D1), 3 (D3), and 7 (D7), visualized via fluorescent microscopy using DiOC6(3)/PI staining: cytoplasm in green and nuclei in red. Scale bar ∼100 μm.

Next, the morphology of HDFn cells upon incubation with conductive fibers’ extracts was visualized using light microscopy (Figure 8) and fluorescent microscopy (Figure 9), where cytoplasm and nuclei labeling (DiOC6(3)/PI) was employed. Cells cultured in the presence of Gr/PLCL polymer showed morphology comparable to that of control cells (untreated) on all experimental days. Moreover, a visually notable increase in confluency from Day 1 to Day 3 supports reported metabolic and proliferation activity data. In the case of Gr/PLCL_PEDOT:PSS_, light microscopy observation was compromised due to dark clusters present in all tested days. We observed what appeared to be the same clusters at DiOC6(3)/PI staining (Figure 9), where the clusters were stained by DiOC6(3) (green). Despite the occurrence of the clusters, the cells were increasing in confluency during the experiment and their morphology did not suggest any abnormalities. Lastly, cells incubated with Gr/PLCL_Gr.ink_ polymer’s extract exhibited normal morphology and an increasing confluency throughout the experiment, though mild in nature compared to the control. This observation agreed with the previous data where metabolic activity and proliferation rates were less noteworthy for this sample when compared to the control and other tested materials [82–84].

**FIGURE 10:**
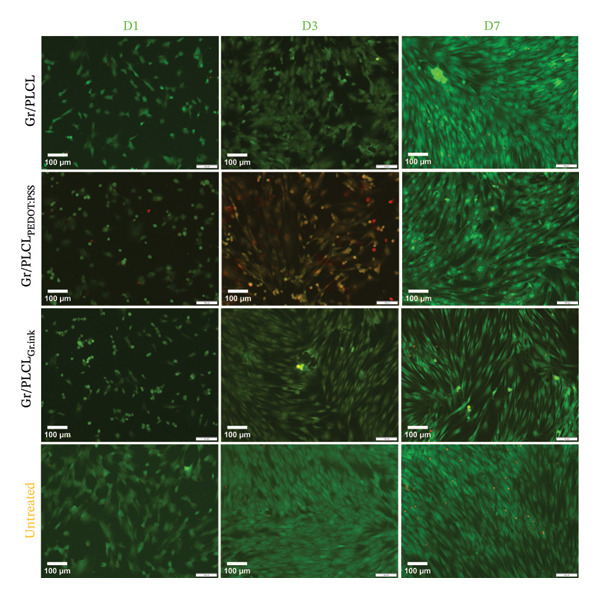
Viability of HDFn cells cultured either in standard conditions (untreated) or incubated with conductive fiber mats’ extracts for 1 (D1), 3 (D3), or 7 (D7) days, visualized via fluorescent microscopy; live cells were stained with BCECF‐AM (green) and dead cells with PI (red). Scale bar ∼100 μm.

The viability of HDFn cells cultured either in standard conditions or in the presence of tested materials’ extracts was evaluated next via live/dead staining, i.e., the cells were incubated with BCECF‐AM dye (green) staining live cells only and with PI (red) penetrating the nuclei of none but dead cells (Figure 10). For Gr/PLCL and Gr/PLCL_Gr.ink_ polymers, the cell viability appeared comparable to the control, with no apparent dead cells on any of the experimental days. As for the Gr/PLCL_PEDOT:PSS_ fibers, live/dead staining suggested an increased number of dead cells in culture on Day 3, while on Days 1 and 7, the culture appeared comparable to the control and other materials. This observation aligns with previous studies showing that the biocompatibility of PEDOT:PSS depends on its dose and administration manner [78–81].

Lastly, the conductive fiber mats were tested for their ability to support cell adhesion. As observed both by metabolic activity (Figure 11(a)) as well as dsDNA quantification (Figure 11(b)), the conductive fiber mats did not exhibit adhesion‐supportive qualities: cells seeded directly onto them showed lower metabolic activity on nearly all experimental days except for the Gr/PLCL copolymer on Day 1 post‐seeding (Figure 11(a)). The data on proliferation rate supported this with significantly lower rates for all fiber mats compared to cells seeded on tissue culture plastic (untreated cells), already on Day 1 post‐seeding (Figure 11(b)). Experimental day 3 post‐seeding did not show any significant difference between the control cells vs. cells seeded on fiber mats due to a high standard deviation of the control. On Day 7 post‐seeding, a lower proliferation rate was again observed with cells seeded on materials, with the exception being Gr/PLCL polymer with an increased (though insignificant) proliferation rate compared to control, untreated cells. Overall, tested copolymers exhibited little to no ability to adhere to HDFn cells. This is not surprising due to the hydrophobic nature of graphene [91], as well as PLA and PCL, forming the PLCL copolymer [92–94]. These findings are consistent with previous studies on graphene‐containing scaffolds, which have shown varied effects on cell metabolic activity in different tissue engineering applications [71–77].

FIGURE 11Metabolic (a) and proliferation activity (b) of HDFn cells seeded and cultured on the conductive fiber mats 1 (D1), 3 (D3), and 7 (D7) days after the cells were seeded. The results were compared to the cells seeded on tissue culture plastic (untreated cells) using one‐way ANOVA with Tukey post hoc test, ^∗∗^ *p* ≤ 0.01, ^∗∗∗^ *p* ≤ 0.001, and ^∗∗∗∗^ *p* ≤ 0.0001.

**Figure:**
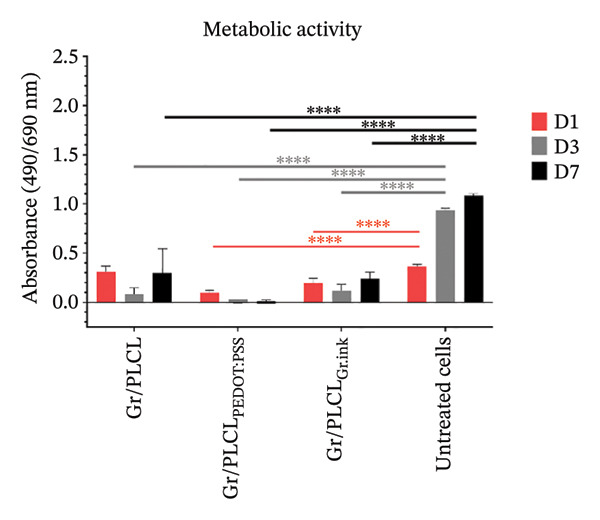
(a)

**Figure:**
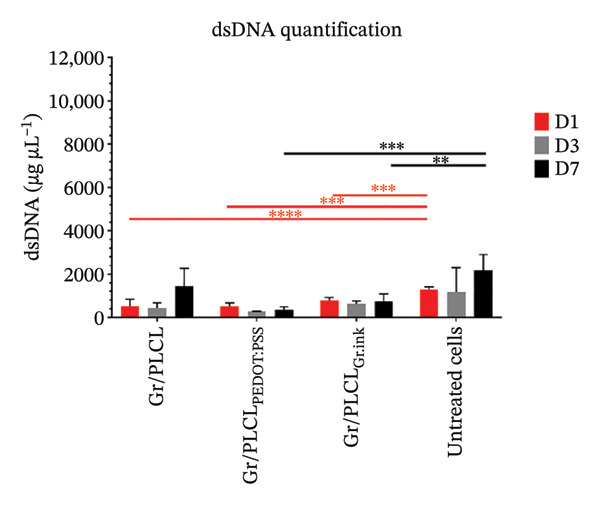
(b)

All tested materials exhibited limited cell adhesion. Cell adhesion is influenced not only by chemical composition, which alters charge, surface energy, contact angle, and subsequently the adsorption of cell‐adhesion mediating proteins, such as vitronectin or fibronectin, from the medium. In addition, surface topography and roughness, as well as mechanical properties, influence cell adhesion, proliferation, and differentiation [95, 96]. As described in the results section, the base copolymer PLCL/graphene (PLCL/Gr) is highly hydrophobic, which complies with the low cell attachment observed [95, 97, 98]. Although the PEDOT:PSS coating reduced the contact angle (82.5 ± 10°) and the graphene‐ink coating even resulted in an extremely hydrophilic behavior, neither of these modifications improved cell adhesion in our setting. This may be explained by a low adsorption of proteins from the medium on highly hydrophilic surfaces [95]. Wettability alone thus seemed not to be the dominant factor in terms of cell adhesion [99, 100]. For PEDOT:PSS coating, though it accounted for improved conductivity and lower hydrophobicity relative to uncoated PLCL/Gr, the relatively smooth and chemically inert surface probably could not accommodate integrin‐mediated cell attachment due to the lack of needed biochemical motifs [101, 102]. Regarding the graphene‐ink coating exhibiting superhydrophilicity, such rapid water‐spreading suggests unfavorable protein adsorption, potentially hindering the formation of stable focal adhesions [103]. To improve cytocompatibility, we propose functionalizing the base polymer (e.g., via introducing polar functional groups, thus making the base copolymer less hydrophobic); coating with bioactive proteins (e.g., fibronectin and vitronectin) or ECM‐like peptides (e.g., RGD) or considering the composite conductive and adhesion‐accommodating coating (e.g., PEDOT:PSS + RGD proteins), and lastly, considering surface micropatterning or roughening of the polymer surface.

Overall, the cytotoxicity/biocompatibility studies presented here suggest that the tested copolymers are promising candidate materials for implantation devices, as evidenced by reported in vitro data using human dermal fibroblasts. Regarding the conductivity under biological conditions, in our setup, the conductivity and short‐circuit current were characterized prior to cell seeding. Thus, cell proliferation could not influence triboelectric performance. In future studies, we aim to assess electrical output under cell‐populated and dynamic conditions, as these might influence charge transfer efficiency.

## 4. Conclusion

Bioresorbable and biocompatible polymer PLCL has been successfully electrospun and processed to obtain a bioelectrode for integration into triboelectric nanogenerators (TENGs). The addition of graphene XT3 to the nanofibers by blending was performed within the matrix, and a further inclusion of the conducting materials on the surface of the fibers was performed by dip coating and brushing. SEM analysis revealed that the coating of the conducting materials has minimal interference with the morphology of nanofibers. The mechanical and thermal analyses have shown an improvement in the physicochemical properties of the PLCL nanofibers after coating the fibers with graphene ink and PEDOT:PSS. Moreover, the electrical properties have been improved significantly. The analysis and comparison of nanofibers before and after coating via infrared spectroscopy confirm the successful deposition of the coating materials on the surface of the nanofibers. Furthermore, biological analyses of nanofibers revealed that they possess significant biocompatibility. However, further improvements, especially in electrical properties, are necessary to increase their attractiveness compared to the metallic electrodes, for instance, by increasing the thickness of depositing materials or by including intermediate molecules to adhere a larger amount of conducting material on the surface of the fibers, as well as providing additional stability. Overall, this study demonstrates a distinct electrode concept for biodegradable TENGs: a fully polymeric, metal‐free, and bioresorbable nanofiber mat with dual conductivity enhancement that provides mechanical compliance, cytocompatibility, and capability for use in a triboelectric device. This combination differentiates our approach from earlier biodegradable electrodes that commonly rely on metallic conductors or structurally rigid composites. However, this work has demonstrated a platform and pathway to achieve stable nanostructured bioelectrodes for many applications where minimal cytotoxicity is the precedence. Further work is in motion to boost specifically the electrical properties of the nanostructured bioelectrodes, which will broaden the usage of such platforms beyond TENGs to a wide range of self‐powered biomedical and miniaturized devices.

## Author Contributions

Viraj P. Nirwan and Viktorie Ročková: writing–review and editing the original draft, methodology, investigation, and formal analysis. Altangerel Amarjargal and Rebecca Hengsbach: writing–review and editing, investigation, and formal analysis. Martin Timusk: writing–review and editing, supervision, funding acquisition, and conceptualization. Linards Lapčinskis: review and editing the original draft, methodology, investigation, and formal analysis. Eva Filová: conceptualization, writing–review and editing, formal analysis, supervision, and funding acquisition. Andris Šutka and Amir Fahmi: writing–review and editing, supervision, funding acquisition, and conceptualization.

## Funding

This research was financially supported mainly by ERA.Net Plus (Project Bioresorbable Implantable Triboelectric Nanogenerator Devices, “BIOTENG”) and partially supported by the European Regional Development Fund–Excellent Research in Regenerative Medicine (No. CZ.02.01.01/00/22_008/0004562).

Open Access funding enabled and organized by Projekt DEAL.

## Ethics Statement

The study was conducted in accordance with the Declaration of Helsinki and approved by the Institutional Ethics Committee of the Institute of Experimental Medicine of the Czech Academy of Sciences (protocol code 2024/4).

## Conflicts of Interest

The authors declare no conflicts of interest.

## Supporting Information

Supporting Information includes the following figures and tables, as mentioned in the manuscript, in the sequence of their occurrence in the text. Figure S1: tensile test analysis of nanofiber bioelectrodes. Figure S2: gradient calculation after tensile test analysis of nanofiber bioelectrodes to determine the Young’s modulus of the respective sample. Figure S3: voltage measurements at (A) 10 GΩ, (B) 1 GΩ, (C) 550 MΩ, (D) 300 MΩ, (E) 200 MΩ, (F) 100 MΩ, (G) 90 MΩ, (H) 80 MΩ, (I) 70 MΩ, (J) 60 MΩ, (K) 50 MΩ, and (L) 10 MΩ load resistances for the TENG device based on PGS and PLA contact layers with Gr/PLCL_PEDOT:PSS_ as electrodes. Table S1: TGA data of pristine and functionalized nanofibers highlighting the main thermal events. Figure S4: TGA (a) shows mass degradation (%) with respect to increasing temperature, and DTG (b) highlights main weight loss events and degradation behavior of the pristine PLCL and hybrid fibers containing conducting functional elements, graphene XT3, PEDOT:PSS, and graphene ink immobilized by blending and deposition, respectively. Figure S5: DSC analysis of the functionalized and coated fibers showed a similar thermal behavior when compared to pristine PLCL nanofibers, with a small increment in their melting endotherm peak temperatures: (a) DSC curve obtained after subjecting the fibers to the first heating cycle and (b) thermal behavior shown by the samples after exposing them to the second heating cycle in DSC; ^∗^melting peak temperature (*T*_*m*_) and glass transition temperature (*T*_*g*_). Table S2: results of the DSC analysis indicate major phase changes observed in the samples and their corresponding temperatures. Figure S6: voltage as a function of load resistance. Figure S7: voltage discharge curves for 470 nF capacitor with connected (a) 10 MΩ, (b) 50 MΩ, (c) 80 MΩ, and (d) 100 MΩ resistances and (e) without additional external load. Table S3: experimental and theoretical time constants for a 470 nF capacitor discharged through different resistances.

## Supporting information


**Supporting Information** Additional supporting information can be found online in the Supporting Information section.

## Data Availability

The data that support the findings of this study are available from the corresponding author upon reasonable request.
