# Supplementary material for: Advanced Flexible Bioelectrodes for Next‐Generation Implantable Triboelectric Nanogenerators
Source: Int J Biomater. 2026 Apr 27;2026:5695793. doi: 10.1155/ijbm/5695793 (PMC13121566; doi:10.1155/ijbm/5695793)
Supplement: Supplementary file 1 — Supporting Information Additional supporting information can be found online in the Supporting Information section. [file IJBM-2026-5695793-s001.docx]

**Supplementary Information**

**Advanced Flexible Bioelectrodes for Next-Generation Implantable Triboelectric Nanogenerators**

*Viraj P. Nirwan^1^, Altangerel Amarjargal^1^, Viktorie Ročková^2,5^, Martin Timusk^3^, Linards Lapčinskis^4^, Rebecca Hengsbach^1^, Eva Filová^2^, Andris Šutka^4^*** and Amir Fahmi^1^**

^1^Rhine-Waal University of Applied Sciences, Faculty of Technology and Bionics, Marie-Curie-Straße 1, 47533 Kleve, Germany;

^2^Institute of Experimental Medicine of the Czech Academy of Sciences, Vídeňská 1083, 14220 Prague 4, Czechia;

^3^Institute of Physics, University of Tartu, W. Ostwaldi Str. 1, 50411 Tartu, Estonia;

^4^Institute of Materials and Surface Engineering, Faculty of Materials Science and Applied Chemistry, Riga Technical University, P. Valdena Street 3, LV1048 Riga, Latvia;

^5^Department of Physiology, Faculty of Science, Charles University in Prague, Viničná 7, 12800 Praha 2, Czechia;

E-Mail: [amir.fahmi@hochschule-rhein-waal.de](mailto:amir.fahmi@hochschule-rhein-waal.de)

Keywords: electrospun hybrid nanofibers, triboelectric nanogenerators, biocompatible polymers, TENG-powered capacitors.


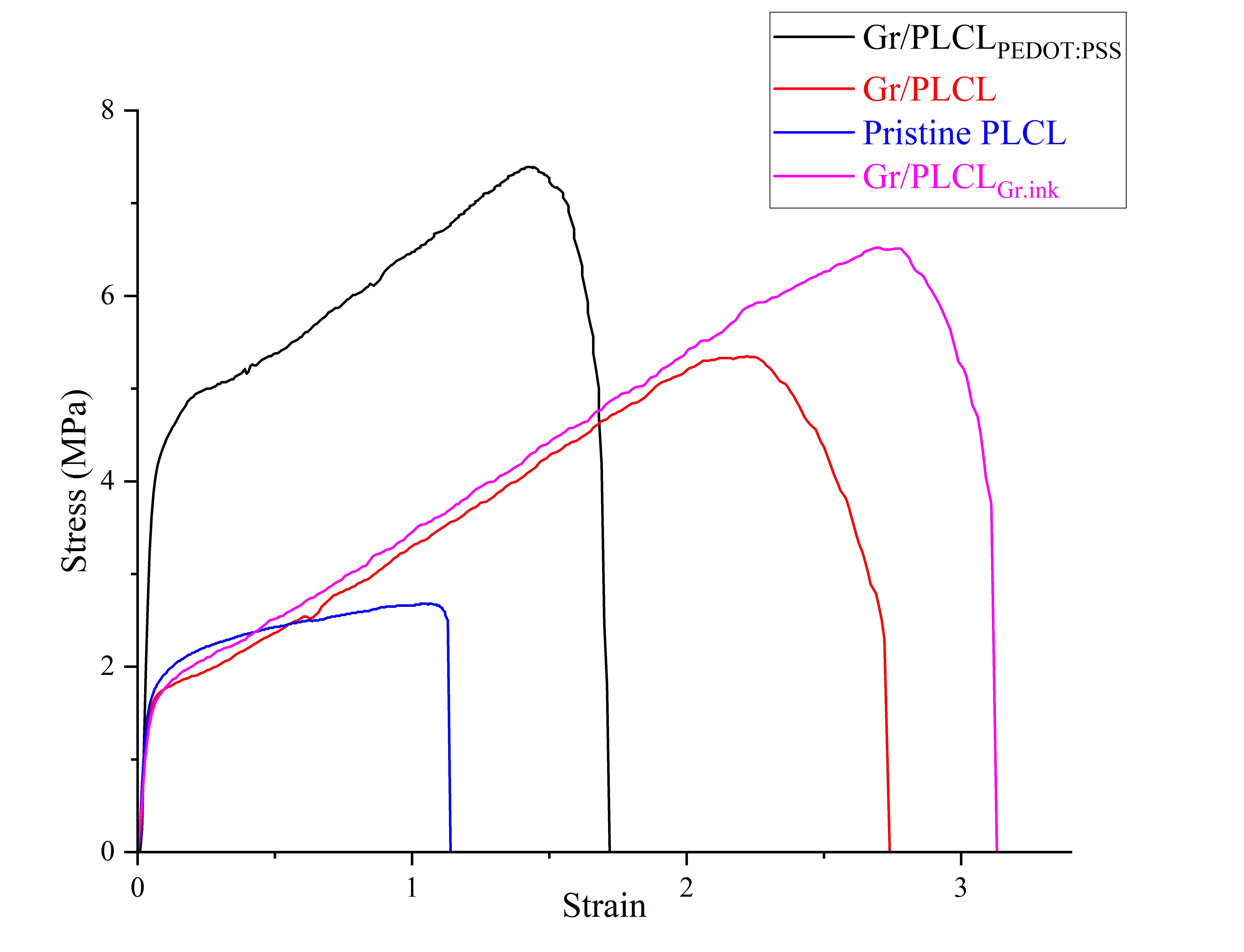


**Figure S1.** Tensile test analysis of nanofiber bioelectrodes.


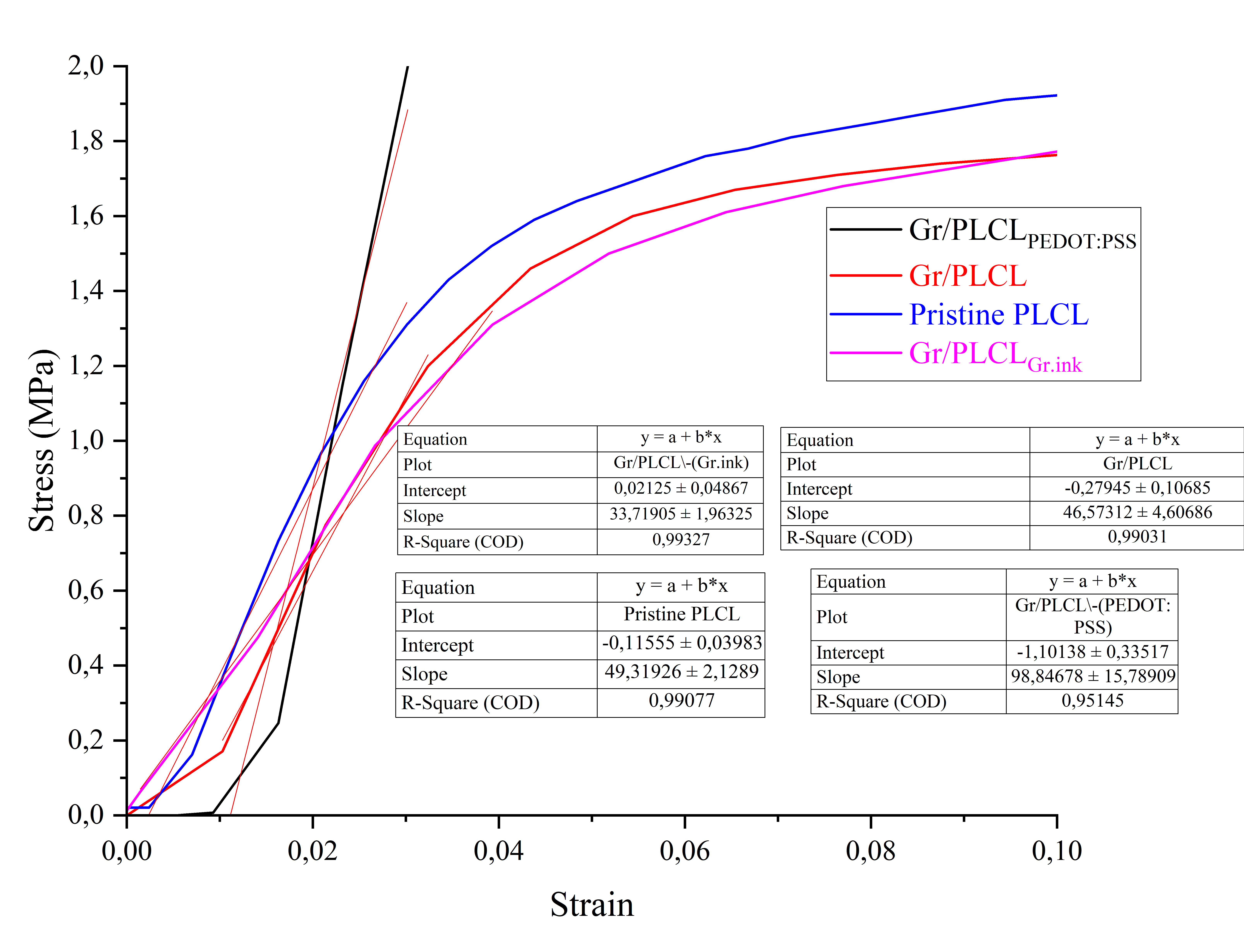


**Figure S2.** Gradient calculation after tensile test analysis of nanofiber bioelectrodes to determine the Young’s modulus of the respective sample.


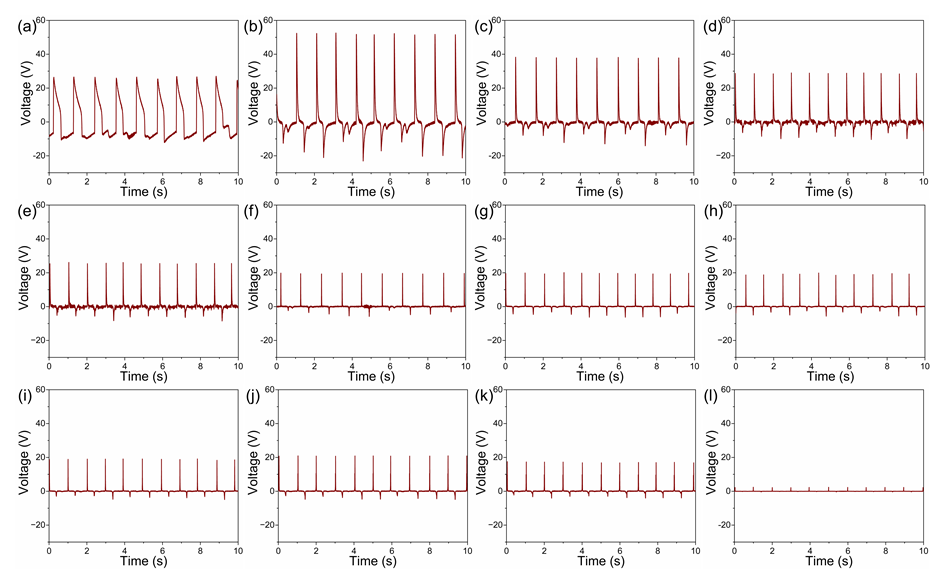


**Figure S3.** Voltage measurements at A) 10 GΩ, B) 1 GΩ, C) 550 MΩ, D) 300 MΩ, E) 200 MΩ, F) 100 MΩ, G) 90 MΩ, H) 80 MΩ, I) 70 MΩ, J) 60 MΩ, K) 50 MΩ and L) 10 MΩ load resistances for TENG device based on PGS and PLA contact layers with Gr/PLCL_PEDOT:PSS_ as electrodes.

**Table S1.** TGA data of pristine and functionalized nanofibers highlighting the main thermal events.

| Sample name | T_Onset_ (°C) | T_Endpoint_ (°C) | T_Inflection point_ (°C) | Weight loss (%) |
| --- | --- | --- | --- | --- |
| Pristine PLCL | 298 | 420 | 333 | 97.6 |
| Gr/PLCL | 289 | 439 | 320 | 92.7 |
| Gr/PLCL_PEDOT:PSS_ | 356 | 444 | 378 | 84 |
| Gr/PLCL_Gr.ink_ | 288 | 453 | 316 | 89 |


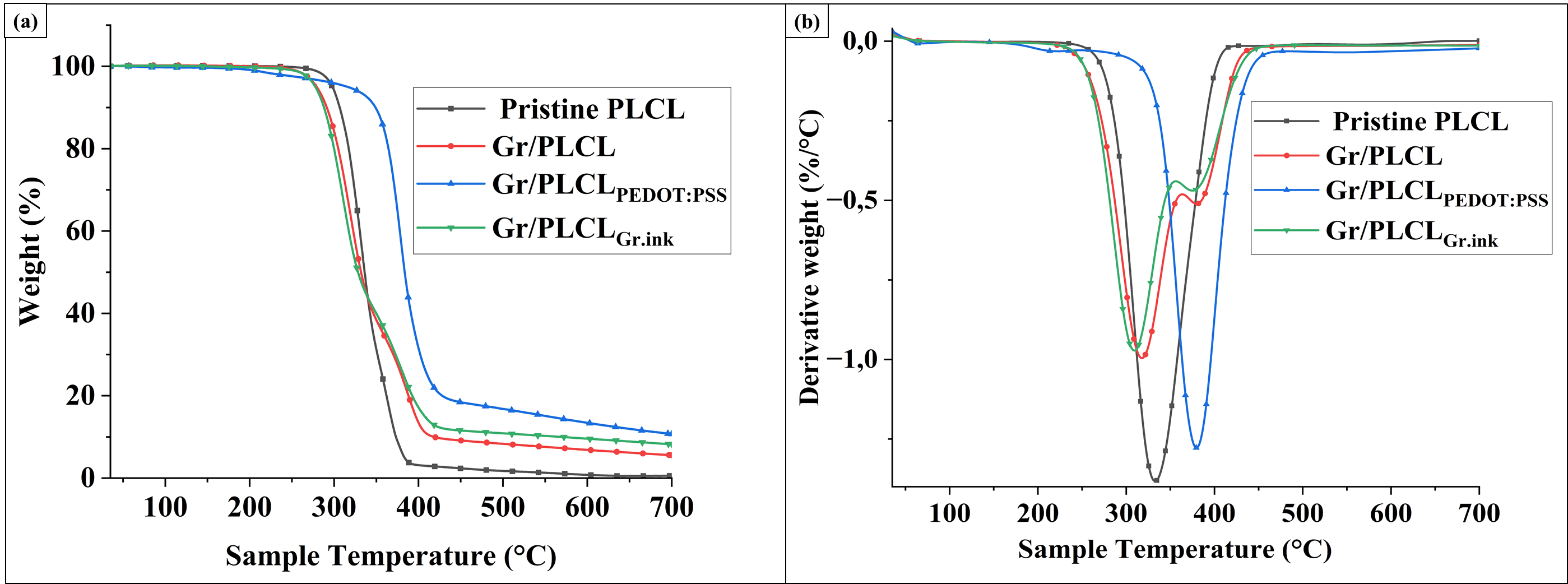


**Figure S4.** TGA (a) shows mass degradation (%) with respect to increasing temperature, and DTG (b) highlights main weight loss events and degradation behavior of the pristine PLCL and hybrid fibers containing conducting functional elements, graphene XT3, PEDOT:PSS, graphene ink immobilized by blending and deposition, respectively.


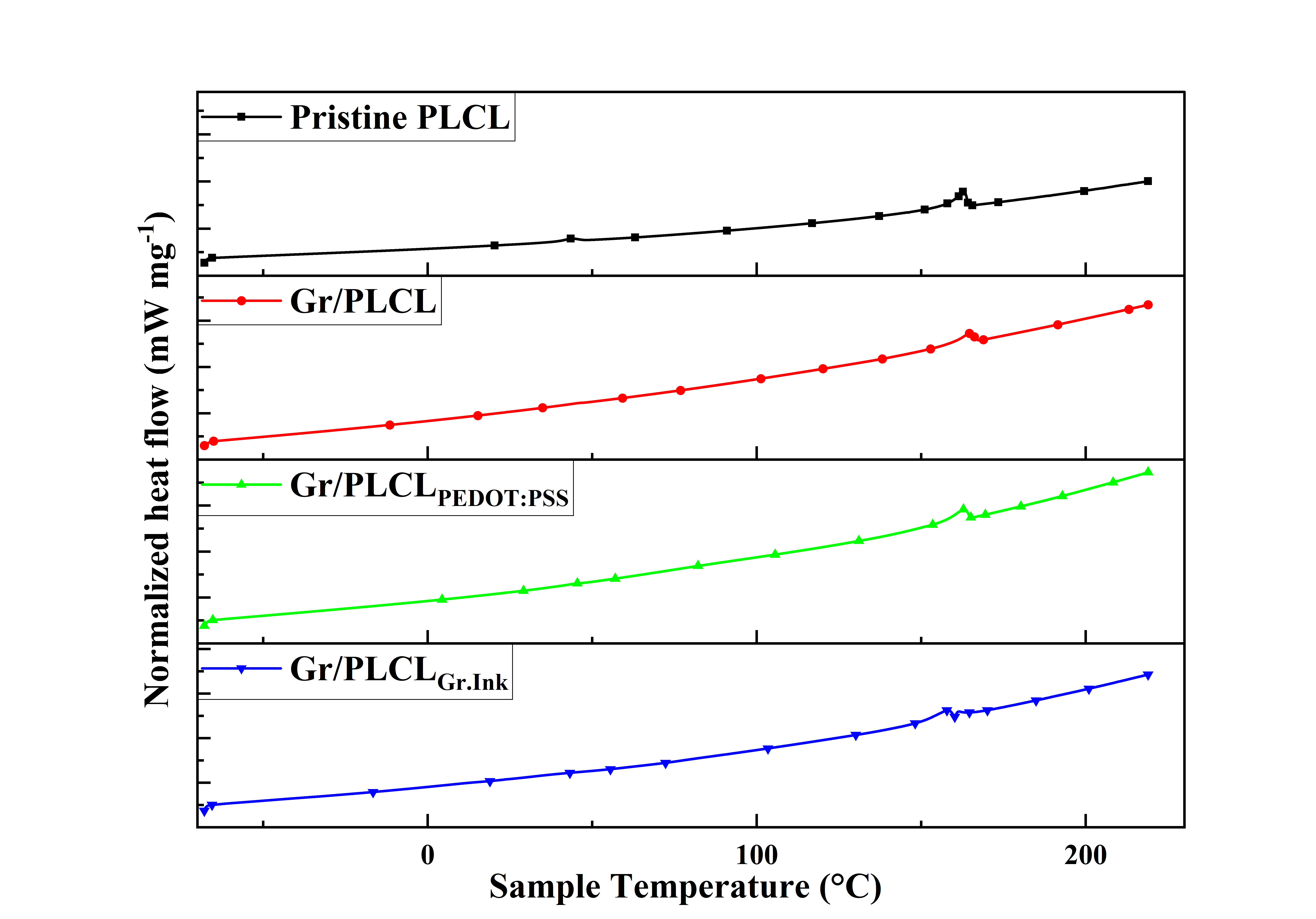

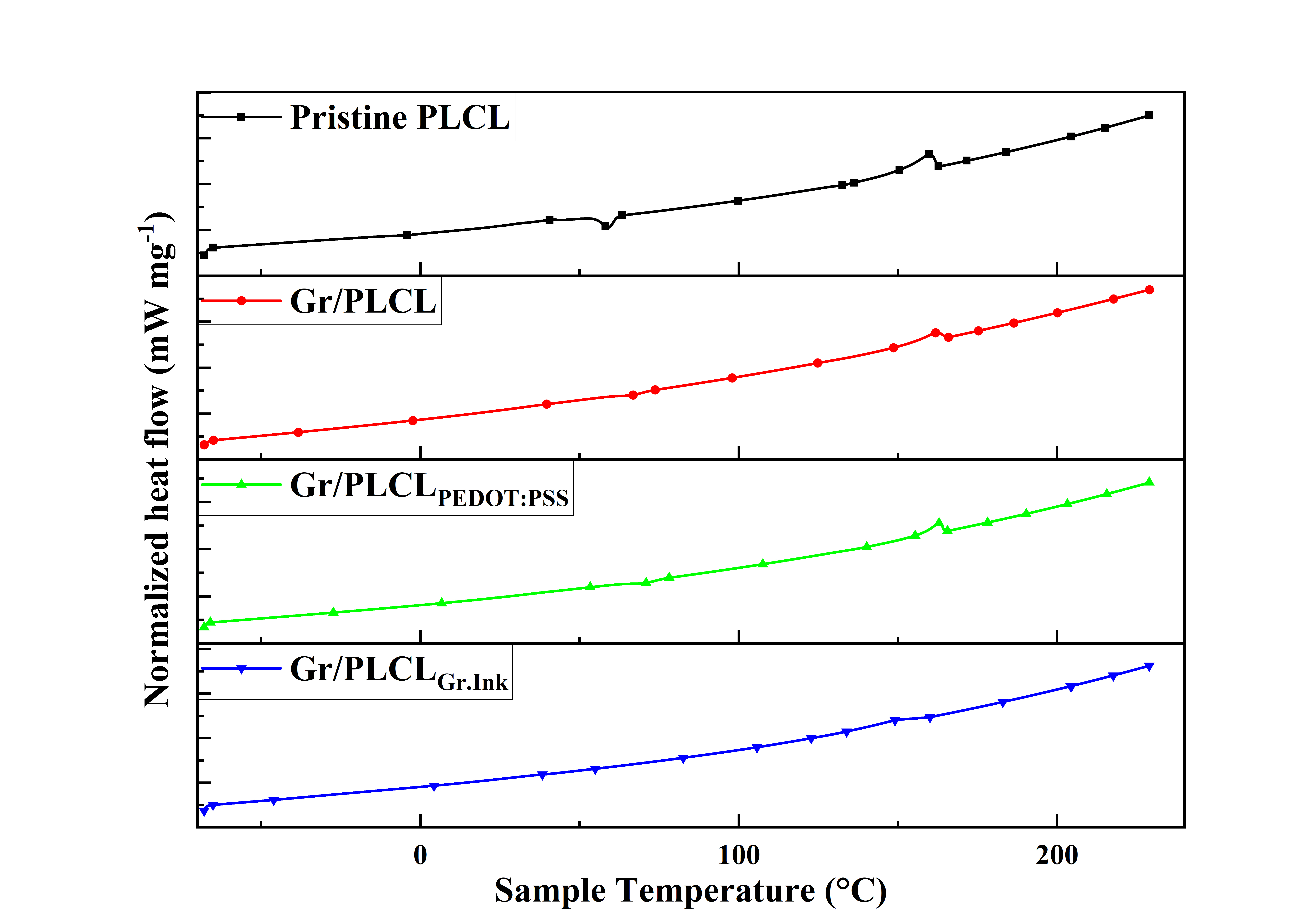


**(a)**

**(b)**

**Figure S5.** DSC analysis of the functionalized and coated fibers showed a similar thermal behavior when compared to pristine PLCL nanofibers with a small increment in their melting endotherm peak temperatures. a) DSC curve obtained after subjecting the fibers to the first heating cycle, b) thermal behavior shown by the samples after exposing them to the second heating cycle in DSC. *melting peak temperature (T_m_), glass transition temperature (T_g_)

**Table S2.** Results of the DSC analysis indicate major phase changes observed in the samples and their corresponding temperatures.

| Sample name | 1^st^ heating scan | | 2^nd^ heating scan | |
| --- | --- | --- | --- | --- |
|  | T_g_ (°C) | T_m_ (°C) | T_g_ (°C) | T_m_ (°C) |
| Pristine PLCL | 39 | 162 | 42 | 159 |
| Gr/PLCL | 44 | 164 | 59 | 162 |
| Gr/PLCL_PEDOT:PSS_ | 45 | 163 | 60 | 163 |
| Gr/PLCL_Gr.ink_ | 41 | 158 | 38 | 149 |



**Figure S6.** Voltage as a function of load resistance.


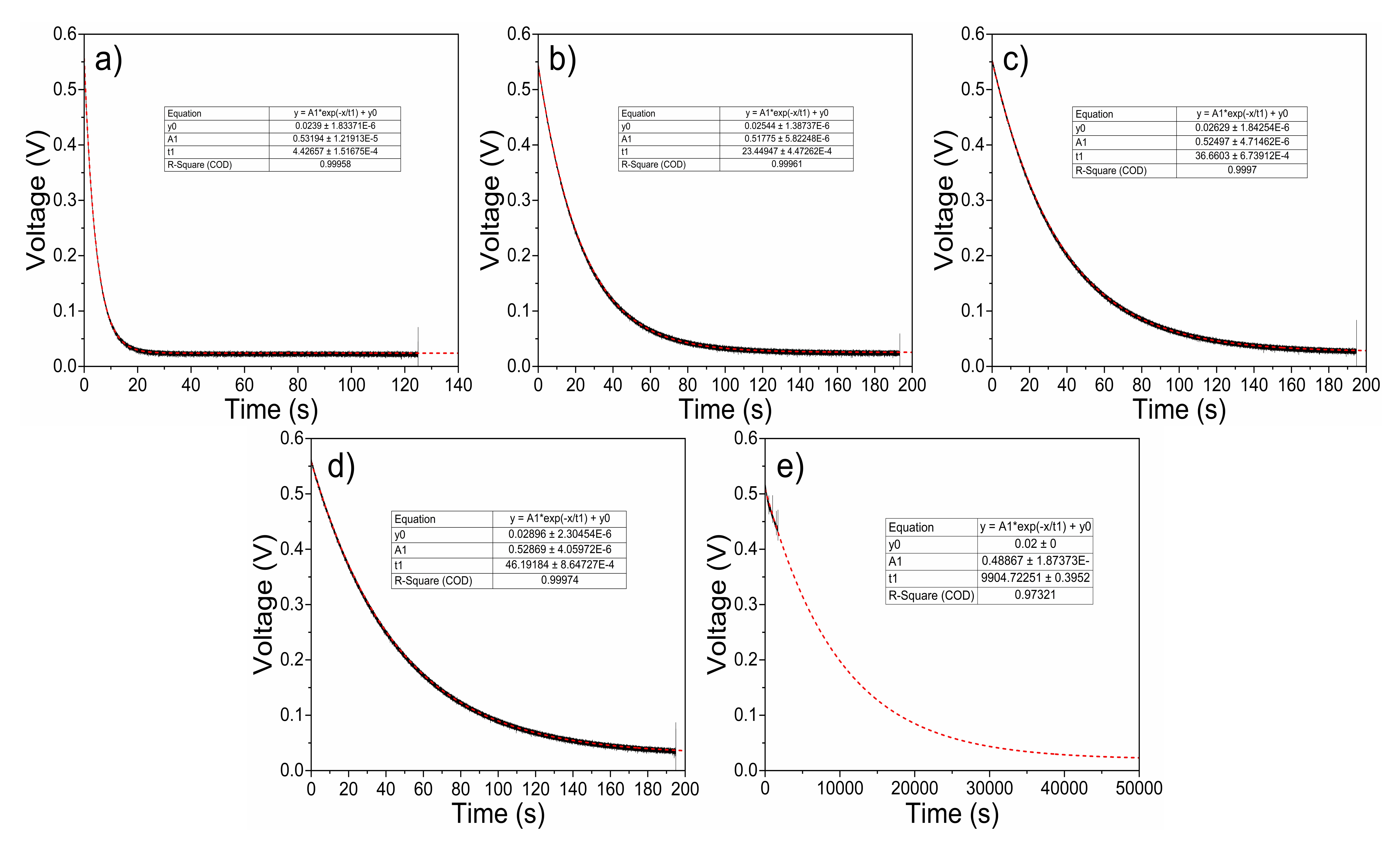


**Figure S7.** Voltage discharge curves for 470 nF capacitor with connected a) 10 MΩ, b) 50 MΩ, c) 80 MΩ, d) 100 MΩ resistances, and e) without additional external load.

**Table S3.** Experimental and theoretical time constants for a 470 nF capacitor discharged through different resistances.

| Resistance | τ_exp_, s | τ_theor_, s |
| --- | --- | --- |
| 10 MΩ | 4.4 ± 0.0 | 4.7 |
| 50 MΩ | 23.4 ± 0.0 | 23.5 |
| 80 MΩ | 36.7 ± 0.0 | 37.6 |
| 100 MΩ | 46.2 ± 0.0 | 47.0 |
| Self-discharge | 9904.7 ± 0.4 | - |
